# Regulating intracellular fate of siRNA by endoplasmic reticulum membrane-decorated hybrid nanoplexes

**DOI:** 10.1038/s41467-019-10562-w

**Published:** 2019-06-20

**Authors:** Chong Qiu, Hu-Hu Han, Jing Sun, Hai-Tao Zhang, Wei Wei, Shi-He Cui, Xin Chen, Jian-Cheng Wang, Qiang Zhang

**Affiliations:** 1 Beijing Key Laboratory of Molecular Pharmaceutics and New Drug Delivery Systems, State Key Laboratory of Natural and Biomimetic Drugs, School of Pharmaceutical Sciences, Peking University, 100191 Beijing, China; 2 Xiangya School of Pharmaceutical Sciences, Central South University, 410013 Changsha, China

**Keywords:** Biotechnology, Cancer, Cell biology

## Abstract

Most cationic vectors are difficult to avoid the fate of small interfering RNA (siRNA) degradation following the endosome-lysosome pathway during siRNA transfection. In this study, the endoplasmic reticulum (ER) membrane isolated from cancer cells was used to fabricate an integrative hybrid nanoplexes (EhCv/siRNA NPs) for improving siRNA transfection. Compared to the undecorated Cv/siEGFR NPs, the ER membrane-decorated EhCv/siRNA NPs exhibits a significantly higher gene silencing effect of siRNA in vitro and a better antitumor activity in nude mice bearing MCF-7 human breast tumor in vivo. Further mechanistic studies demonstrate that functional proteins on the ER membrane plays important roles on improving cellular uptake and altering intracellular trafficking pathway of siRNA. It is worth to believe that the ER membrane decoration on nanoplexes can effectively transport siRNA through the endosome-Golgi-ER pathway to evade lysosomal degradation and enhance the silencing effects of siRNA.

## Abstract (web summary)

The silencing efficiency of siRNA delivered by non-viral cationic vectors is limited due to endosomal/lysosomal degradation. Here, the authors develop endoplasmic reticulum-coated vesicles as a biomimetic vector for siRNA delivery and show that they improve inhibition of tumor growth in mice in vivo.

## Introduction

In the past years, many non-viral cationic vectors such as liposomes, polymers, and polymeric micelles have been used to overcome extracellular and intracellular obstacles during small interfering RNA (siRNA) transfection^1–3^. However, most of these cationic vectors were difficult to avoid the fate of endosomal/lysosomal degradation following clathrin-mediated endocytosis (CME), meaning that most of siRNAs were damaged by the acidic enzyme-rich environments in endosomes/lysosomes^4, 5^. Delivered by such a tragic intracellular trafficking pathway of endosome–lysosome, the gene silencing effects of siRNA is naturally weakened. Contrastingly, it is noted that the route of endosome–Golgi–endoplasmic reticulum (ER) was exploited as a safe trafficking pathway for drug transportation, such as cholera toxin and some viruses mediated drug delivery systems^6–10^. The strategy of altering intracellular trafficking pathway provides a promising insight into the development of siRNA delivery systems.

The cell-membrane-modified nanoparticles by utilization of natural cellular membranes, including platelet (PLT), red blood cell (RBC), leukocyte, cancer cell, macrophage, and mesenchymal stem cell, have been developed as biomimetic drug delivery systems^11–20^. These membrane-coated biomimetic nanoparticles were mainly used for targeted drug delivery to primary tumors or other diseases based on the unique function of cell membrane surface antigen. As the largest intracellular endomembrane system, ER membrane (EM) has not been fully recognized and used as a modifier of biomimetic drug delivery system. Various resident proteins in EM could induce the directional transportation between Golgi apparatus and ER by COPI or COPII vesicles^21–25^. Compared to the aforementioned endosome–lysosome pathway, this endosome–Golgi apparatus/ER pathway will be beneficial for transporting siRNA-loaded vectors to ER through Golgi apparatus via a COPI-mediated retrograde transport pathway, avoiding the lysosomal degradation pathway.

**Fig. 1:**
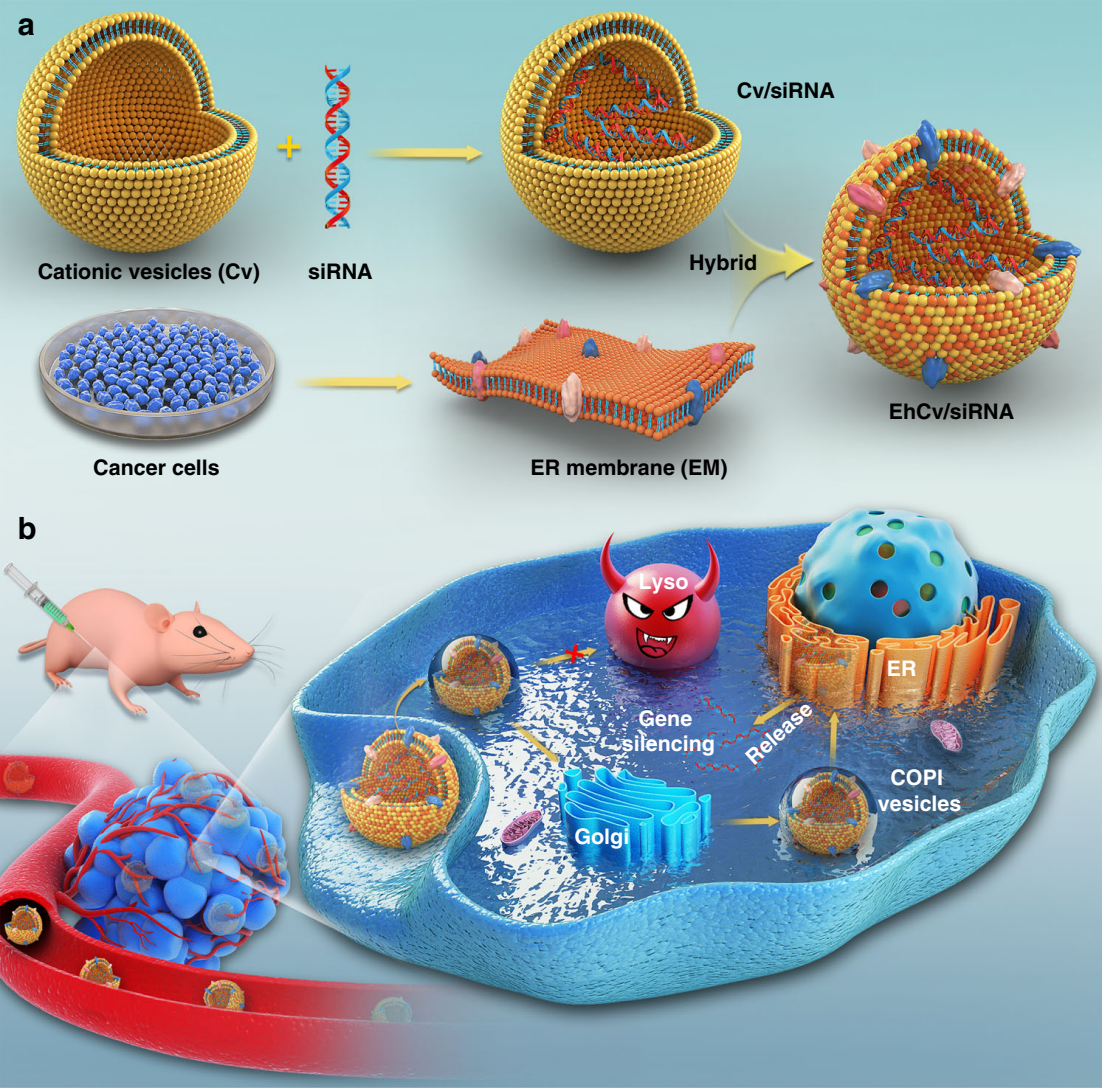
The assembly process and delivery of ER membrane (EM)-hybrid small interfering RNA (siRNA)-loaded nanoplexes (EhCv/siRNA NPs). **a** The EhCv/siRNA NPs were prepared with the decoration of cancer cell EM on Cv/siRNA NPs through membrane fusion; **b** EhCv/siRNA NPs were injected peritumorally to MCF-7 tumor in situ, then it would promote cell internalization and transport siRNA to the ER via a COPI-mediated retrograde Golgi–ER pathway instead of endosome–lysosome pathway, so as to evade intracellular lysosomal degradation and enhance siRNA silencing effects

Herein an EM-decorated hybrid nanoparticle (EhCv/siRNA NPs) is developed in this study to improve siRNA transfection through regulating intracellular fate of siRNA. The endogenous EM resourced from cancer cells is isolated for the modification of siRNA-loaded vectors. It is hypothesized that the decoration of EM on siRNA-loaded lipoplexes (Cv/siRNA NPs) could promote cell internalization and transport siRNA to the ER via COPI vesicles, so as to evade intracellular lysosomal degradation and enhance siRNA silencing effects (Fig. 1). The integrative nanostructure, intracellular trafficking pathway, gene silencing capability, and tumor therapeutic efficiency of EhCv/siRNA NPs are characterized and evaluated in vitro and in vivo.

## Results

### Preparation and characterization of EhCv/siRNA NPs

The EM-hybrid nanoplexes (EhCv/siRNA NPs) were prepared with the following three steps: (i) EM was isolated from cancer cells, (ii) preparing siRNA-loaded cationic vehicles (Cv/siRNA), and (iii) decorating Cv/siRNA lipoplexes with EM by ultrasonic dispersion (Fig. 1a). As seen in Fig. 1a, EM was isolated from human cancer cells^26^. The Cv prepared with the optimal ratio (1:1:1, *n*/*n*/*n*) of 1,2-dioleoyl-3-trimethylammonium-propane (DOTAP), 1,2-dioleoyl-sn-glycero-3-phoshoethanolamine (DOPE), and cholesterol compacted negative siRNA to form Cv/siRNA NPs at the N/P ratio of 5/1 with an electrostatic interaction (Supplementary Fig. 1). Finally, the membrane-hybrid cationic vehicles (EhCv) were prepared by the insertion of EM on the Cv/siRNA NPs with lipid membrane fusion at a weight ratio of EM to Cv (0.5/1, w/w) under ultrasonic method^27–29^. In addition, the cell membrane–hybrid nanoplexes (ChCv/siRNA NPs) as a control formulation was prepared with cancer cell membrane (CM) instead of EM by the methods as described in the preparation of EhCv/siRNA NPs (Supplementary Fig. 1).

**Fig. 2:**
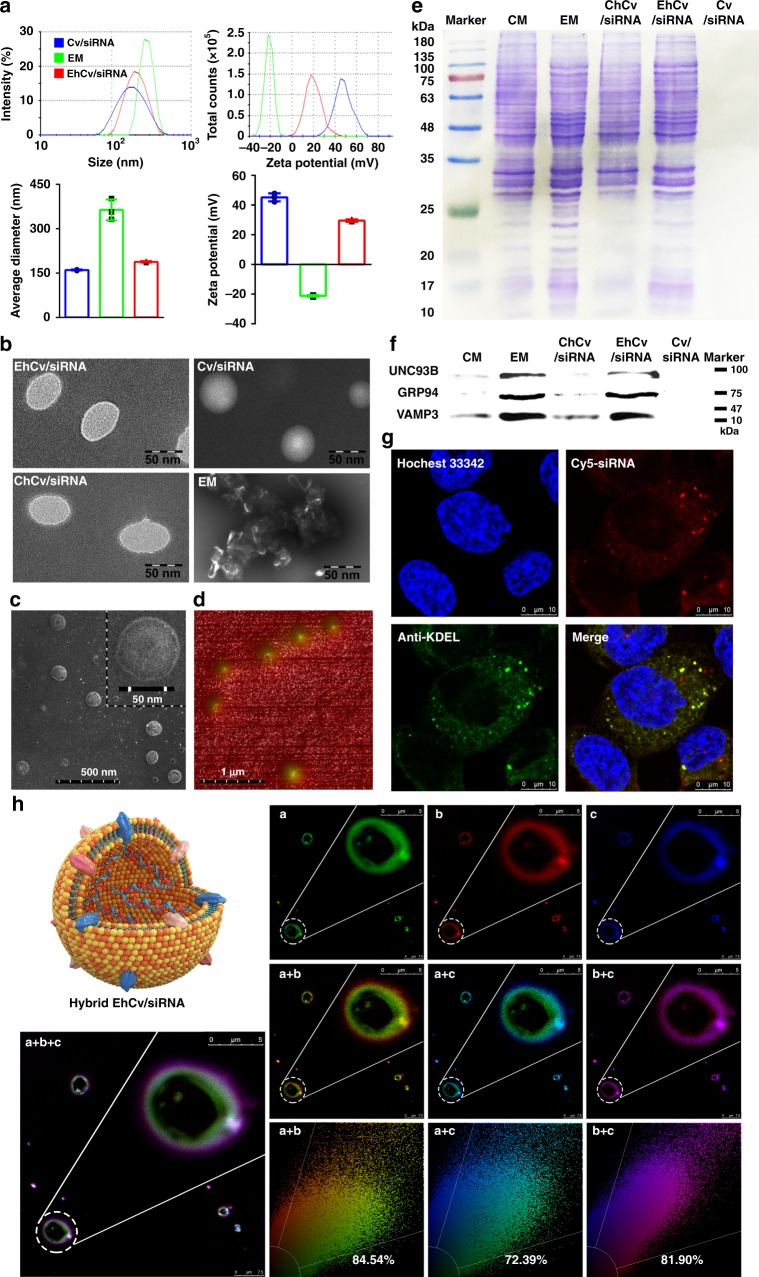
Characterization of ER membrane (EM)-hybrid small interfering RNA (siRNA)-loaded nanoplexes (EhCv/siRNA NPs). **a** Particle sizes and zeta potentials of the Cv/siRNA NPs, ER membrane (EM), and EhCv/siRNA NPs. **b** Transmission electron microscopic images of various NPs. **c** Scanning electron microscopic images of EhCv/siRNA NPs. **d** Atomic force microscopic images of EhCv/siRNA NPs. **e** Sodium dodecyl sulfate-polyacrylamide gel electrophoresis and **f** western blot analysis of membrane proteins. **g** Intracellular fluorescence colocalization of EM (green) and Cv/siRNA NPs (red) detected by confocal laser scanning microscopic (CLSM) imaging. **h** Simulation analysis of integrative structure of EhCv/siRNA NPs detected by CLSM, in which the EM, Cv, and siRNA were labeled by Alexa Fluor 488-labeled Anti-KDEL antibody (a, green), rhodamine-DOPE (b, red), and Cy5 (c, blue), respectively. The colocalization ratio was calculated by Pearson’s correlation coefficient

After EM decoration, the hydrodynamic diameter of EhCv/siRNA NPs was 188.2 ± 2.6 nm (mean ± SD, *n* = 3), slightly bigger than that of Cv/siRNA NPs (160.4 ± 1.9 nm) (Fig. 2a and Supplementary Table 1). The images of scanning electron microscope (SEM) and atomic force microscope (AFM) indicated that the EhCv/siRNA NPs were in spherical shape with good monodispersity, and particle size measured by SEM or AFM was smaller than the hydrodynamic diameter (Fig. 2a, c, d), which attributed to dehydration of the hydration layer. In addition, the zeta potentials of the EhCv/siRNA NPs were 29.5 ± 0.8 mV, significantly lower than that of Cv/siRNA NPs (45.1 ± 2.7 mV) (Fig. 2a and Supplementary Table 1). The obvious lower zeta potential found in the EhCv/siRNA NPs was resulted from the insertion of negative-charged EM (−21.2 ± 0.6 mV) through lipid membrane fusion. The images of SEM and transmission electron microscope (TEM) showed that the clear and bright circular boundary displayed around the surface of EhCv/siRNA NPs as well as seen from ChCv/siRNA NPs (Fig. 2b, c), which probably attributed to the enhanced brightness of membrane protein staining with phosphotungstic acid during sample preparation^30^.

To confirm the integrative structure of EhCv/siRNA NPs with the decoration of EM is necessary for next biological evaluation. First, the protein components in the EM and EhCv/siRNA NPs were analyzed by sodium dodecyl sulfate (SDS)-polyacrylamide gel electrophoresis and western blotting assay, respectively. As seen in Fig. 2e, the primary protein components from the EM mostly remained in EhCv/siRNA NPs, especially for UNC93B^31^ and GRP94^32, 33^ as two important protein markers of EM (Fig. 2f). Meanwhile, the vesicle-associated membrane protein 3 (VAMP3), one of the SNARE family related to intracellular vesicle trafficking^34, 35^, was detected in EM and EhCv/siRNA NPs. Similarly, the protein components retained in ChCv/siRNA NPs were consistent with the electrophoresis strips of cell membrane, but no protein signal was detected in Cv/siRNA NPs without membrane decoration. Second, a higher intracellular fluorescence colocalization rate (87.83 ± 5.08%) observed from the dual-labeled EhCv/siRNA NPs with anti-KDEL antibody^23^ (green) and Cy5-siRNA (red) implied that the internalized EhCv/siRNA NPs could keep structural integrity well after endocytosis for a certain period (Fig. 2g). Third, the assembly mechanism of hybrid EhCv/siRNA NPs was confirmed by the fluorescence colocalization between EM (green), Cv (red), and siRNA (blue), which were marked by Alexa Fluor^@^488-labeled anti-KDEL antibody, rhodamine, and Cy5, respectively. Before ultrasonic mixing, the confocal laser scanning microscopic (CLSM) images showed that EM vesicles were almost adsorbed around Cv vesicles with an electrostatic interaction and the fluorescence colocalization (yellow) between EM and Cv was only 39.56% (Supplementary Fig. 2). As shown in Fig. 2h, with the process of ultrasonic mixing, the aggregation of EM together with Cv vesicles disappeared and changed into the uniformly dispersed vesicles with a higher fluorescence colocalization (yellow, 84.54%), suggesting that most of EM was probably fused with Cv vesicles into hybrid membrane layer by lipid arrangement under ultrasonic mixing. Meanwhile, the fluorescence colocalization (cyan) between EM and siRNA increased from 12.41% to 72.39% under ultrasonic mixing, although the fluorescence colocalization of Cv and siRNA did not show significant change (83.23% vs 81.90%) before and after ultrasonic mixing. Collectively, these abovementioned evidences demonstrated that the EM-decorated EhCv/siRNA NPs were successfully prepared as an integrative hybrid nanostructure.

In addition, the results of hemolysis assay, cytotoxicity, and gel retardation assay demonstrated that the EhCv/siRNA NPs had a better safety and stability than Cv/siRNA NPs (Supplementary Fig. 3), suggesting that EM-decorated EhCv/siRNA NPs would be a safe delivery system for in vivo application.

### In vitro gene silencing effects of EhCv/siRNA NPs

**Fig. 3:**
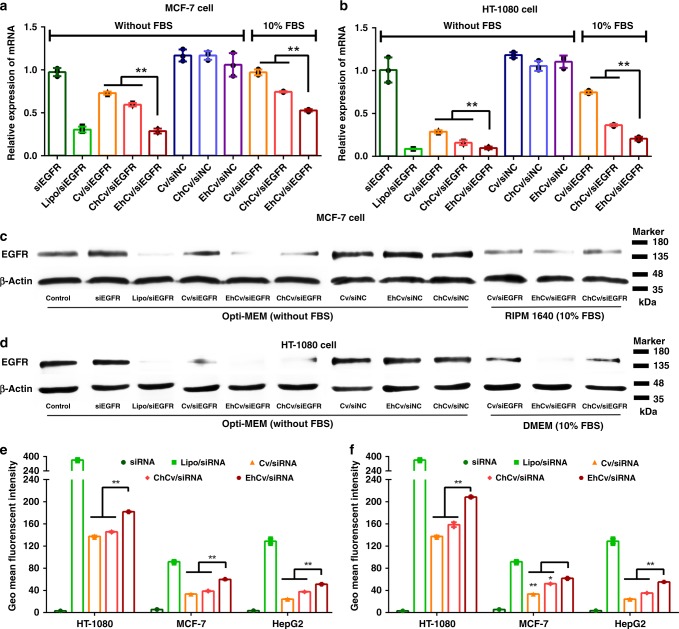
The gene silencing effect and cellular uptake of various small interfering RNA (siRNA)-loaded nanoparticles (NPs). **a**, **b** Relative levels of epidermal growth factor receptor (EGFR) mRNA on MCF-7 cells and HT-1080 cells detected by reverse transcriptase-PCR, respectively. **c**, **d** Expression levels of EGFR protein on MCF-7 cells and HT-1080 cells detected by western blot assay, respectively. **e**, **f** Intracellular fluorescence intensities on MCF-7, HT-1080, and HepG2 cells detected by flow cytometry (FAM-labeled siNC: 100 nM). The final concentration of siRNA was 100 nM. Data are shown as mean ± SD (n = 3). *p < 0.05, **p < 0.01, analysis of variance

The effects of EM decoration on improving gene silencing of the EhCv/siEGFR NPs were investigated on MCF-7 cancer cells. Epidermal growth factor receptor (EGFR)-targeted gene downregulation is effective for cancer therapy. In Supplementary Fig. 4, it was shown that the IC_50_ value of EhCv/siEGFR NPs (~50 nM) was significantly lower than that of ChCv/siEGFR NPs (~160 nM) and Cv/siEGFR NPs (~200 nM) (*p* < 0.01, *t* test), suggesting that the EM-decorated EhCv/siEGFR NPs could exhibit a stronger gene silencing effect. At the siEGFR concentration of 100 nM, the EM-decorated EhCv/siEGFR NPs could exhibit about two-folds higher downregulation of EGFR mRNA (~75%) on MCF-7 cells in Opti-MEM without fetal bovine serum (FBS), compared to ChCv/siEGFR NPs (~40% downregulation) and Cv/siEGFR NPs (~30% downregulation), (Fig. 3a), although a relatively higher zeta potential was observed in the Cv/siEGFR NPs (Fig. 2a and Supplementary Table 1). As shown in Fig. 3c, the distinct EGFR protein bands were observed in ChCv/siEGFR NPs and Cv/siEGFR NPs, whereas EhCv/siEGFR NPs presented almost invisible protein band. These significant effects on the downregulation of protein expression and EGFR mRNA induced by EhCv/siEGFR NPs demonstrated that EM decoration played an important role on improving gene silencing of siRNA. Apart from EM derived from MCF-7 cancer cells, another EM isolated from HT-1080 cancer cells was used to fabricate EhCv/siEGFR NPs, which also exhibited significant higher gene silencing effects than HT-1080 CM-decorated ChCv/siEGFR NPs and Cv/siEGFR NPs (Figs. 3b, d). Generally, transfection efficiency of siRNA was always interfered by the negatively charged serum because of the instability of nanoplexes and the formation of protein corona during delivery in circulation^36^. Interestingly, our results demonstrated that EhCv/siEGR NPs could exhibit a significant higher downregulation on EGFR mRNA and protein expression than ChCv/siEGFR NPs and Cv/siEGFR NPs when transfected in medium containing 10% FBS. Therefore, the enhanced gene silencing effects of EhCv/siEGFR NPs were probably attributed to the improved cellular uptake and the changes of intracellular trafficking pathway of siRNA nanoplexes by the modification of EM decoration.

### Cellular uptake of EhCv/siRNA NPs

**Fig. 4:**
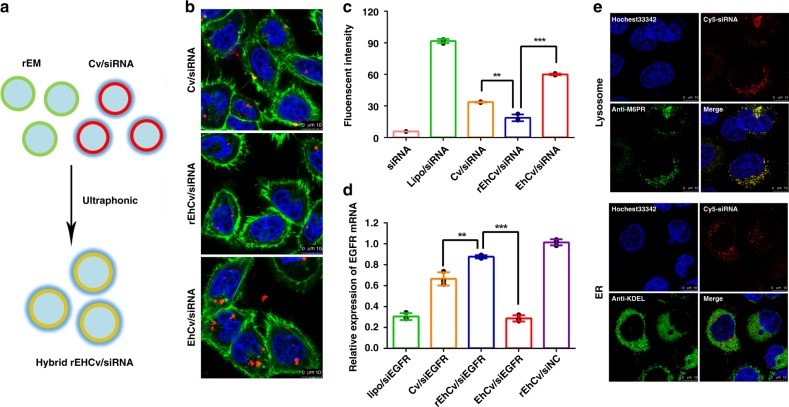
Functional verification of protein-removed endoplasmic reticulum (ER) membrane (rEM). **a** The rEhCv/siRNA NPs was prepared with rEM whose proteins were removed; **b** Intracellular fluorescence detected by confocal microscopy; **c** Intracellular fluorescence intensities detected by flow cytometry; **d** Relative levels of epidermal growth factor receptor (EGFR) mRNA detected by reverse transcriptase-PCR; **e** Intracellular fluorescence colocalization of rEhCv/siRNA NPs in ER and lysosomes, which were stained by Alexa Fluor^@^568-labeled anti-KDEL and anti^@^M6PR antibody, respectively. The final concentration of FAM-siNC, Cy5-siNC, and siEGFR were 100 nM. Data are shown as mean ± SD (*n* = 3). **p < 0.01, ***p < 0.001, *t* test

The effects of EM decoration on improving cellular uptake of the EhCv/siEGFR NPs were investigated on MCF-7, HT-1080, and HepG2 cancer cells, respectively. As shown in Fig. 3e, the EhCv/siEGFR NPs with a decoration of EM derived from MCF-7 cells could exhibit higher intracellular fluorescence intensities than ChCv/siEGFR NPs and Cv/siEGFR NPs in all three cell models. It was suggested that decoration of EM derived from MCF-7 cells could enhance cellular uptake ability of EhCv/siEGFR NPs in three different types of cells. Similarly, the EhCv/siEGFR NPs with a decoration of EM isolated from HT-1080 cells also exhibited a relative higher cell uptake with no cell type selectivity (Fig. 3f). The fact that the decoration of EM improving cell uptake of EhCv/siEGFR NPs in the corresponding cancer cells was further confirmed by different fluorescence intensities of CLSM images (Supplementary Figs. 5 and 6). Although Cv/siEGFR NPs had a relatively higher positive charge, EhCv/siEGFR NPs still exhibited significantly higher intracellular fluorescence intensity and gene silencing effect on MCF-7 cells (Fig. 3a, e) in a positive-charge-independent manner. To further certify the key function of EM proteins, the proteins were removed from EM by 100 mM sodium carbonate^37^ and the incomplete EM was named as rEM. The incomplete EM-decorated rEhCv/siEGFR NPs (Fig. 4a) showed similar hydrodynamic diameter and zeta potential (197.0 ± 11.7 nm, 30.4 ± 4.1 mV) with the EhCv/siRNA NPs (Supplementary Fig. 7a and Supplementary Table 1). However, different from the complete EM-decorated EhCv/siRNA (Fig. 2b), the images of TEM indicated that the rEhCv/siRNA NPs did not show the clear and bright circular boundary around outer of EhCv/siRNA NPs (Supplementary Fig. 7b), which attributed to the loss of membrane proteins^30^. The absence of membrane proteins was further validated in the lysate of rEhCv/siRNA NPs and rEM by gel electrophoresis and western blot assay (Supplementary Fig. 7c, d). More interestingly, compared to those of intact EM-decorated EhCv/siEGFR NPs, the intracellular fluorescence intensity (Fig. 4b, c) and the gene silencing capacity (Fig. 4d) of incomplete EM-decorated rEhCv/siEGFR NPs were dramatically decreased. The significant difference between rEhCv/siEGFR and EhCv/siEGFR implied that those proteins expressed in EM probably played important roles in promoting cell uptake of NPs. It was reported that the SNARE proteins expressed in EM^38^ could mediate the movement of cholesterol or lipids and thus resulting in a following membrane fusion immediately^39, 40^. Compared to ChCv/siRNA NPs, the EhCv/siRNA NPs possessed more SNARE proteins (10–30 kDa, Fig. 2e), in which some proteins (such as VAMP3 (Fig. 2f)) plays an important role for membrane binding and fusion^34, 35^. Therefore, it is reasonable to believe that the decoration of EM on siRNA-loaded cationic vehicle is probably a suitable strategy that can be used to effectively improve cellular uptake.

### Intracellular distribution and trafficking pathway of EhCv/siRNA NPs

The effects of EM decoration on altering intracellular trafficking pathway of EhCv/siEGFR NPs were also investigated on MCF-7 cancer cells. To a certain extent, the internalization fate of siRNA-loaded NPs was often affected by the endocytosis pathways including clathrin-mediated endocytosis (CME), caveolae-mediated endocytosis (CeME), and macropinocytosis (MP)^41^. To verify the internalization pathway of EhCv/siRNA NPs, three kinds of green fluorescence markers including Alexa 488-Tf, Alexa 488-CTB, and fluorescein isothiocyanate (FITC)-dextran were applied to label the corresponding channels of CME, CeME, and MP, respectively. As seen from Supplementary Fig. 8, the internalization of EhCv/siRNA NPs was mainly dependent on the CeME pathway compared to those of ChCv/siRNA NPs and Cv/siRNA NPs, indicating that the decoration of EM was beneficial to the enhanced CeME pathway of EhCv/siRNA NPs. It has been reported that SNARE proteins (such as VAMP3) are related to the intracellular vesicular transport, which could ferry cargo from CeME-related endosomes to *trans*-Golgi network^42^. Therefore, it is not hard to understand that the enhanced CeME pathway of EhCv/siRNA NPs probably attributed to the decoration of EM membrane with SNARE protein expression.

**Fig. 5:**
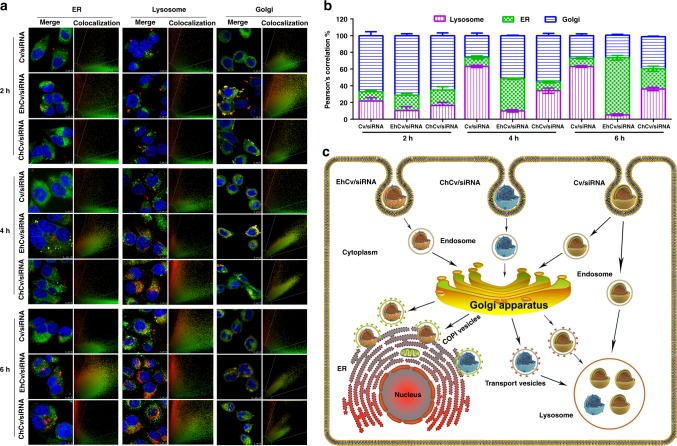
Intracellular trafficking pathways of various small interfering RNA (siRNA)-loaded nanoparticles (NPs) on MCF-7 cells. **a** Representative colocalization images of different NPs in endoplasmic reticulum (ER), lysosomes, and Golgi apparatus at different times detected by confocal laser scanning microscope. The yellow dots represent the colocalization of fluorescence-labeled organelles (green) and Cy5-siRNA (red). **b** Quantity analysis of intracellular colocalization ratios of different NPs in ER, lysosomes, and Golgi at different times. The colocalization ratios were calculated based on the Pearson’s correlation coefficient from multiple images (*n* = 3) with a normalized calculation method. **c** Schematic illustration of intracellular trafficking pathways of different NPs

After endocytosis, the intracellular fates of NPs were usually dependent on the different trafficking pathways, such as endosome–lysosome and endosome–Golgi–ER^6–10, 43^. To further distinguish the different trafficking pathways of EhCv/siRNA, ChCv/siRNA, and Cv/siRNA NPs, the CLSM images of the corresponding organelles were captured (Fig. 5a) and the fluorescence colocalization ratios were calculated from the Pearson’s correlation coefficient (Fig. 5b). Obviously, three NPs were approximate 70% colocalization ratios of fluorescence distribution with Golgi within initial 2 h after incubating with MCF-7 cells. Subsequently, the fluorescence colocalization ratios of EhCv/siRNA NPs in Golgi gradually decreased to ~50% (4 h) and ~25% (6 h), whereas in ER were increased to ~40% (4 h) and ~70% (6 h). In contrast, the fluorescence colocalization ratios of ChCv/siRNA NPs were of ~10% at 4 h and ~25% at 6 h and those of Cv/siRNA NPs were of ~10% at 4 and 6 h. It indicated that EhCv/siRNA NPs had a significant higher ER retention behavior than ChCv/siRNA and Cv/siRNA NPs. More interestingly, the fluorescence colocalization ratios of EhCv/siRNA NPs in lysosomes were of ~10% (4 h) and ~5% (6 h), which were remarkably lower than those of ChCv/siRNA NPs (~35% at 4 h and ~40% at 6 h) and Cv/siRNA NPs (~65% at 4 and 6 h). These results demonstrated that EhCv/siRNA NPs did transport siRNA through the endosome–Golgi–ER pathway.

### Intracellular trafficking mechanisms of EhCv/siRNA NPs

**Fig. 6:**
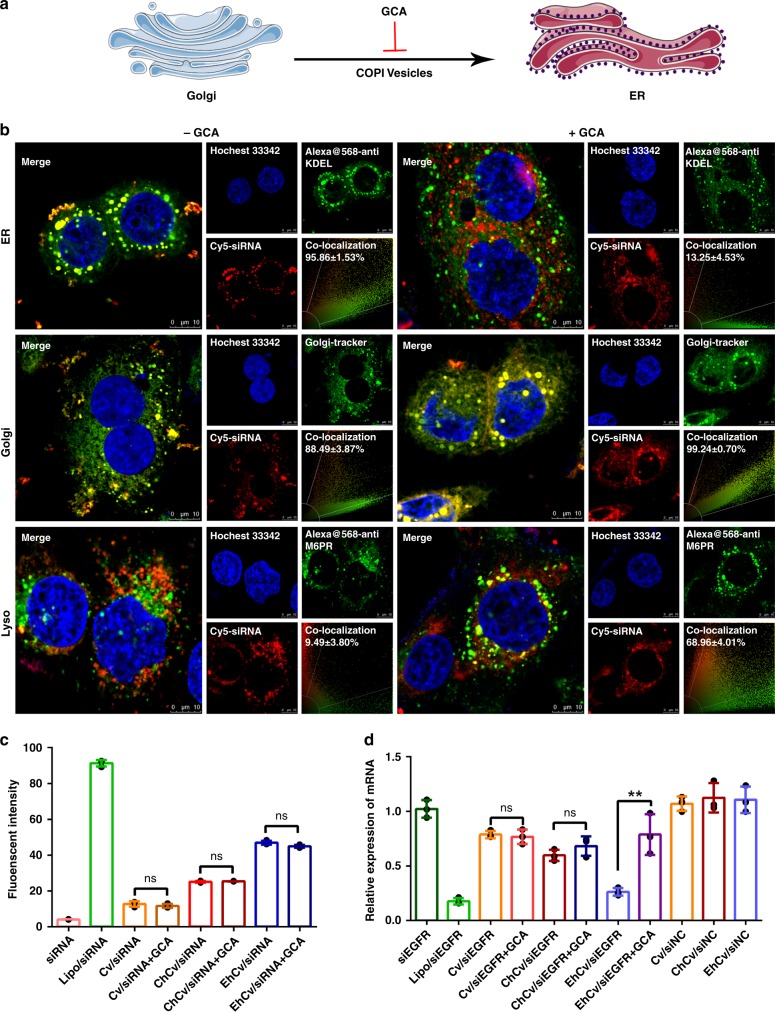
Verification of the retrograde transport pathway of EhCv/siRNA NPs from Golgi to endoplasmic reticulum (ER): **a** Illustration of Golgicide A (GCA) inhibition; **b** Representative colocalization pictures of EhCv/siRNA NPs in ER, Golgi apparatus, and lysosomes with or without GCA treatment, **c** Cellular uptake and **d** epidermal growth factor receptor (EGFR) mRNA levels in the cells treated by EhCv/siRNA NPs with or without GCA treatment (10 μM). Golgi, ER, and lysosomes were stained by Golgi-tracker, Alexa Fluor^@^568-labeled anti-KDEL, and anti-M6PR antibody, respectively. The final concentration of FAM-siNC, Cy5-siNC, and siEGFR were 100 nM. Data are shown as mean ± SD (*n* = 3). **p < 0.01, *t* test

Three different experimental approaches were performed to further elucidate the intracellular trafficking mechanisms of EhCv/siRNA NPs after endocytosis, including (1) inhibition of COPI-coated vesicle assembly, (2) cellular behavior of incomplete rEhCv/siRNA NPs, and (3) functional verification of ER resident proteins. First, in order to confirm the role of Golgi apparatus during intracellular trafficking pathway of EhCv/siRNA NPs, Golgicide A (GCA) as a specific inhibitor was used for inhibiting assembly of COPI-coated vesicles^21^ (Fig. 6a). The fluorescence colocalization of EhCv/siRNA NPs in organelles, respectively, was observed by CLSM on MCF-7 cells after incubation with or without GCA. It was shown that the fluorescence colocalization ratio of EhCv/siRNA NPs in ER significantly decreased from 95.86 ± 1.53% (−GCA) to 13.85 ± 4.53% (+GCA), whereas the colocalization ratio in Golgi increased from 88.49 ± 3.87% (−GCA) to 99.24 ± 0.76% (+GCA), and the colocalization ratio in lysosomes increased from 9.49 ± 3.80% (−GCA) to 68.96 ± 4.01% (+GCA) (Fig. 6b). These results suggested that EhCv/siRNA NPs were mainly accumulated in Golgi or lysosomes because the transportation of COPI-coated vesicles from Golgi to ER was inhibited by GCA. Furthermore, a significantly lower gene silencing effect was observed in the cells co-treated with EhCv/siEGFR NPs and GCA, while the silencing effects of Cv/siEGFR and ChCv/siEGFR NPs were not significantly affected by GCA (Fig. 6d). The GCA did not affect the cellular uptake of various NPs (Fig. 6c). These results indicated that transportation from Golgi to ER via COPI vesicles is a critical route of EhCv/siEGFR NPs.

Second, the fluorescence colocalization ratio of incomplete EM-decorated rEhCv/siEGFR NPs in ER and lysosomes were observed by CLSM on MCF-7 cells. The CLSM images displayed that most of rEhCv/siRNA NPs were completely colocalized with lysosomes at 6-h time point, whereas no visible colocalization in ER (Fig. 4e). Obviously, the intracellular fluorescence intensity (Fig. 4b, c) and the gene silencing capacity (Fig. 4d) of incomplete EM-decorated rEhCv/siEGFR NPs were significantly lower than those of intact EM-decorated EhCv/siEGFR NPs. Compared to 10% downregulation of EGFR mRNA by rEhCv/siEGFR NPs, an excellent gene silencing effect (~70% downregulation, *p* < 0.001, analysis of variation (ANOVA)) was observed in EhCv/siEGFR NPs. These results suggested that the ER proteins played an important role in transporting siRNA-loaded nanoplexes from Golgi to ER.

**Fig. 7:**
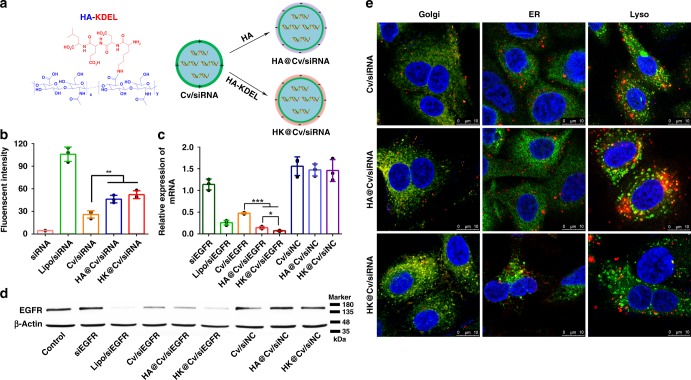
Functional verification of endoplasmic reticulum (ER)-resident proteins. **a** Schematic illustration of the preparation of HA@Cv/siRNA and HK@Cv/siRNA NPs; **b** Intracellular fluorescence intensities in A549 cells detected by flow cytometry; **c** Relative levels of epidermal growth factor receptor (EGFR) mRNA detected by reverse transcriptase-PCR; **d** Expression levels of EGFR protein detected by western blot assay; **e** Intracellular fluorescence colocalization of Cv/siRNA, HA@Cv/siRNA, and HK@Cv/siRNA NPs in Golgi, ER, and lysosomes, which were stained by Golgi-tracker, Alexa Fluor^@^568-labeled anti-KDEL, and anti^@^M6PR antibody, respectively. The final concentration of FAM-siNC, Cy5-siNC, and siEGFR were 100 nM. Data are shown as mean ± SD (*n* = 3). *p < 0.05, **p < 0.01, ***p <0.001, *t* test

In addition, the key role of ER-resident proteins on transporting siRNA-loaded nanoplexes via Golgi-ER trafficking pathway was further investigated. The signal peptide of ER resident proteins (KDEL) was conjugated with hyaluronic acid (HA) to obtain hyaluronan-KDEL polymer (HK) and used for fabricating HK-modified siRNA-loaded vectors (HK@Cv/siRNA NPs) with a better stability and safety (Fig. 7a, Supplementary Figs. 10 and 11). As shown in Fig. 7b, although both HK@Cv/siRNA NPs and HA@Cv/siRNA NPs exhibited a similar higher cellular uptake than Cv/siRNA NPs, the HK@Cv/siEGFR NPs exhibited a significantly higher gene silencing effects (downregulation of protein and mRNA) than HA@Cv/siEGFR NPs and Cv/siEGFR NPs (Fig. 7c, d). Besides, the HK@Cv/siEGFR NPs also obtained a lower fluorescence colocalization in lysosomes but higher colocalization in ER, when compared to HA@Cv/siEGFR NPs and Cv/siEGFR NPs (Fig. 7e). These results demonstrated that the higher gene silencing effects of HK@Cv/siEGFR NPs were resulted from the decoration of KDEL signal peptide. These experimental results proved that the EM bearing KDEL signal peptide played an important role in transporting siRNA-loaded nanoplexes from Golgi to ER. It has been reported that the KDEL signal peptide could bind to KDEL receptor in *cis*-Golgi membrane following recruited ER-resident proteins into retrograde COPI vesicles from Golgi to ER^23^.

Taken together the above results, it was worth believing that the decoration of EM on siRNA-loaded nanoplexes could effectively alter intracellular trafficking pathway to transport siRNA through the endosome–Golgi–ER pathway instead of the endosome–lysosome pathway, thereby evade intracellular lysosomal degradation and facilitate siRNA release on ER where ribosomes (the translation place of mRNA) mainly distributed on or around, and increase the ability of silencing the corresponding mRNA immediately (Fig. 5c).

### In vivo antitumor effects of EhCv/siRNA NPs

**Fig. 8:**
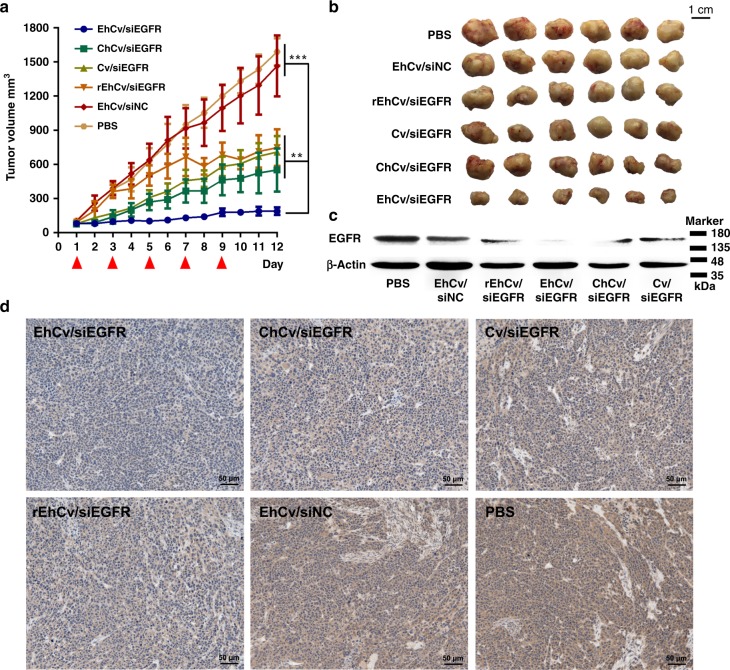
In vivo tumor inhibition effects of various small interfering RNA (siRNA)-loaded nanoparticles (NPs). **a** Tumor growth curves of nude mice (6 mice each group) after treated by various siRNA-loaded NPs (siEGFR or siNC: 0.5 mg kg^−1^) via peritumoral injection. Each dose was indicated by the arrows. **p < 0.01; ***p < 0.001, analysis of variation. **b** Photographs of harvested tumor tissues at the end of 12-day treatment. **c** Western blotting analysis and **d** immunohistochemistry of epidermal growth factor receptor (EGFR) expression in tumor tissues at the end of 12-day treatment

The in vivo antitumor effects of EM-decorated EhCv/siRNA NPs were investigated in nude mice bearing orthotopic MCF-7 breast tumor with a peritumoral administration. The EGFR-targeted siRNA was selected as therapeutic agent. It has been reported that the EGFR is one of the important cytokines and signaling pathways involved in the survival, invasion, and proliferation of tumor cells and the related stromal cells, such as fibroblasts and neovascular endothelial cells^44^. As shown in Fig. 8a, b and Supplementary Fig. 12a, the EM-decorated EhCv/siEGFR NPs revealed the best inhibition of tumor growth (~80%) among all formulations, significantly higher (*p* < 0.01) than those of undecorated Cv/siEGFR NPs (~43%) and CM-decorated ChCv/siEGFR NPs (~0%). However, the incomplete EM-decorated rEhCv/siEGFR NPs only exhibited a lower tumor inhibition (~36%), significantly lower than those of intact EM-decorated EhCv/siEGFR NPs (*p* < 0.01, ANOVA). Meanwhile, the results of western blot and immunohistochemistry (IHC) assay also demonstrated the EhCv/siEGFR NPs could exhibit the best downregulation of EGFR protein in tumor tissues (Fig. 8c, d). The best downregulation of EGFR protein was a convincing evidence to prove that the EhCv/siEGFR NPs exhibited the strongest tumor growth inhibition^45, 46^. The fact that EM-decorated of EhCv/siEGFR NPs could exhibit significantly higher inhibition of tumor growth than other groups enlightened the possible mechanisms: (1) The SNARE proteins (including VAMP3) that existed in EM would benefit to promote membrane fusion with tumor cells and related stromal cells, thereby enhancing cellular uptake of EhCv/siEGFR NPs (Fig. 3c); (2) Some SNARE proteins could mediate the transportation of EhCv/siEGFR NPs from endosomes to Golgi^38^ after cell internalization (Fig. 5a, b); (3) The ER- resident proteins in EhCv/siEGFR NPs activated the retrograde transport pathway from Golgi to ER and avoided the degradation of siEGFR in lysosomes that resulted in a better knockdown effect on EGFR protein and thus powerful inhibition of tumor growth. On the contrary, the ChCv/siEGFR NPs and Cv/siEGFR NPs would be mostly transferred into lysosomes due to lacking of EM proteins. Therefore, we believed that regulating intracellular fate of siRNA by EM-decorated hybrid nanoplexes is a potential strategy for siRNA transfection.

### Safety evaluation of EhCv/siRNA NPs

To evaluate in vivo safety of EhCv/siEGFR NPs, the changes of body weight and important hematological indicators (white blood cell (WBC), RBC, hemoglobin (HGB), and PLT) were quantitatively detected during administration. The pathological sections of main organs (heart, liver, spleen, lung, and kidney) were also detected with a hematoxylin–eosin staining. As shown in Supplementary Fig. 12, it was demonstrated that there was no obvious change of body weight and hematological indicators (WBC, RBC, HGB, and PLT) in nude mice after treatment with EhCv/siEGFR NPs related to other formulations and phosphate-buffered saline (PBS) group, indicating that EhCv/siEGFR NPs did not induce significant adverse effects on immune system and hematological function in nude mice. These results were consistent with the in vitro results of hemolysis assay and cytotoxicity (Supplementary Fig. 3). Besides, compared to other groups, the EhCv/siRNA NPs did not cause obvious pathological changes in main organs (heart, liver, spleen, lung, and kidney) (Supplementary Fig. 13). These results suggested that EhCv/siRNA NPs would be a promising and safe delivery system for in vivo tumor therapy via peritumoral injection.

In summary, this study demonstrated that EM decoration on the siRNA-loaded nanoplexes can significantly improve cellular uptake and gene silencing effects of siRNA by altering intracellular trafficking pathway of EhCv/siEGFR NPs and significantly inhibit tumor cell growth in vitro and in vivo. We believe that utilizing EM-decorated hybrid lipoplexes to regulate intracellular fate of siRNA through the endosome–Golgi–ER instead of endosome–lysosome pathway will become a promising strategy for nucleic acid drug delivery in future.

## Methods

### Materials and reagents

PBS, FBS, OPTI-MEM, and trypsin were purchased from Macgene (Beijing, China). Agarose was obtained from GENE COMPANY (Hong Kong, China); Hoechst 33258, hoechst33342 and Golgi-tracker red were purchased from Molecular Probes Inc. (Oregon, USA); Rhodamine phalloidin, Alexa Fluor 488 conjugated Transferrin, Alexa Fluor 488-Conjugated Cholera Toxin Subunit B and FITC-Dextran were purchased from Invitrogen (Carlsbad, USA); 1,2-dioleoyl-sn-glycero-3-phoshoethanolamine (DOPE), Rhodamine110-DOPE, 1,2-dioleoyl-3-trimethylammonium-propane (DOTAP), (3-(4,5-dimethylthiazolyl-2)-2,5-diphenyltetrazolium bromide) (MTT), and cholesterol were purchased from Sigma Aldrich. Co. (St. Louis, MO). Anti-EGFR siRNA (sense strand: 5′-AGG AAU UAA GAG AAG CAA CAU dTdT-3′ and antisense strand: 5′-AUG UUG CUU CUC UUA AUU CCU dTdT-3′, named as siEGFR), negative control siRNA (sense strand: 5′-UUC UCC GAA CGU GUC ACG UTT-3′; antisense strand: 5′-ACG UGA CAC GUU CGG AGA ATT-3′, named as siNC) and fluorescein-labeled siRNA (5′ end of the sense strand, FAM-siRNA or Cy5-siRNA) were synthesized and purified with HPLC by Gene Pharma Co. Ltd (Shanghai, China). Endoplasmic Reticulum Isolation Kit (ER010) was obtained from Sigma-Aldrich (USA). Alexa Fluor 568-labeled anti-KDEL and anti-M6PR were purchased from Abcam (Shanghai, China). Cell culture reagents and materials were purchased from M&C GENE TECHNOLOGY (Beijing, China).

### Cell lines

Human breast cancer MCF-7 cells, human liver cancer HepG2 cells, human fibrosarcoma cancer HT-1080 cells, and human non-small cell lung cancer A549 cells were provided by the Institute of Basic Medical Science, Chinese Academy of Medical Sciences (Beijing, China). The cells were cultured in the standard cell medium recommended by American Type Culture Collection (ATCC) at 37 °C in a 5% CO_2_ atmosphere.

### Isolation of EM

EM was extracted according to the operation of the Endoplasmic Reticulum Isolation Kit (ER010, Sigma-Aldrich). Briefly, MCF-7, HT-1080 cells were harvested and washed with PBS three times. Then the cells were suspended in the hypotonic extraction buffer (10 mM HEPES, pH 7.8, with 0.25 M sucrose, 1 mM EGTA, and 25 mM potassium chloride) for 20 min at 4 °C. Hereafter, the cells were centrifuged at 600 × *g* for 5 min and mixed with the isotonic extraction buffer (10 mM HEPES, pH 7.8, 1 mM EGTA, and 25 mM potassium chloride) and transferred to a 7 mL Dounce homogenizer. Then the cells were broken with 10 strokes of the Dounce homogenizer and centrifuged at 4 °C (1000 × *g* for 10 min and 12,000 × *g* for 15 min). Subsequently, calcium chloride solution was added into the supernatant fraction with constant stirring. The final concentration of CaCl_2_ was 7 mM and stirred for additional 15 min at 4 °C. Moreover, the sample was centrifuged at 8000 × *g* for 10 min at 4 °C. The pellet was suspended in the isotonic extraction buffer and centrifuged for 60 min at 100,000 × *g* in an ultracentrifuge at 4 °C. Then the supernatant was removed using a pipette. Finally, the ER was collected and the protein concentration was calibrated using the BCA method and stored at −80 °C.

### Extraction of CM

To obtain membrane material, a previously reported extrusion approach was used^17^. MCF-7 and HT-1080 cells were harvested and washed with PBS three times, and the cells that were resuspended in cold Tris buffer (pH = 7.4) containing 10 mM Tris, 10 mM MgCl_2_, and 1 × EDTA-free protease inhibitor were disrupted at 4 °C for 1 h and further homogenized at 10,000 × *g* for 30 min. The homogenized solution was centrifuged at 500 × *g* for 10 min at 4 °C, then the supernatants were centrifuged at 10,000 × *g* for 10 min and further centrifuged at 100,000 × *g* for 1 h. The resulting cell pellet was washed once with 10 mM Tris buffer, centrifuged, resuspended, and sonicated for 5 s using a 4 ultrasonicator (JYD-650L, Zhixin Inc., China). Finally, the CMs were obtained from MCF-7 and HT-1080 cells, and the concentration of membrane protein was calibrated using the BCA method and stored at −80 °C.

### Preparation of EhCv/siRNA NPs

Firstly, the Cvs composed of DOTAP, DOPE, and cholesterol (1:1:1, *n*/*n*/*n*) were prepared by thin film hydration followed by an ultrasonic dispersion and the final concentration of vehicles was 3.1 mg mL^−1^ (5% glucose). Afterwards, 25 μL 5% glucose solution or PBS of siRNA (4 μM) was mixed with Cv to construct Cv/siRNA NPs at different N/P ratios in the same volume, then allowed to incubate for 15 min at 37 °C. The N/P ratio was selected as 5/1 and the Cv/siRNA NPs (total weight concentration: 0.80 mg mL^−1^, siRNA: 2 μM) were obtained. Subsequently, 50 μL Cv/siRNA NPs was dropped into the same volume of EM or CM solution at different weight ratios, followed by a 1-min ultrasonic mixing at room temperature, then the EhCv/siRNA NPs or ChCv/siRNA NPs were prepared, respectively.

### Optimization of EhCv/siRNA NPs

For optimizing the formulation of EhCv/siRNA NPs, the size distribution with various weight ratios of membrane and Cv was detected by a Zetasizer Nano ZS (Malvern, U.K.). Moreover, to investigate the siRNA-loading capability at different weight ratios, the gel retardation assay was conducted. Briefly, the samples were tackled with loading buffer and electrophoresed on a 1% agarose gel containing 0.5 mg mL^−1^ Exred (a special luminant dye for siRNA staining). Electrophoresis was performed at 80 mV for 3 min, subsequently 100 mV for 10 min, and these resulting gels were photographed under ultraviolet (UV) illumination instrument.

In addition, a comparative study on cellular uptake of EhCv/siRNA NPs was conducted in MCF-7 cells. Briefly, the cells were seeded 2.5 × 10^5^ per well in 6-well plates. After 24 h proliferation, those NPs containing FAM-labeled siRNA (final concentration of 100 nM) were exposed to cells for an additional 4 h at 37 °C. After incubation, cancer cells were harvested and washed three times with pre-cooled PBS solution. The intracellular fluorescence intensity of FAM-labeled siRNA was detected by the fluorescence-activated cell sorter (FACS) Calibur flow cytometry (Becton Dickinson, San Jose, CA, USA) immediately at the wavelength of 488 nm.

### Characterization of EhCv/siRNA NPs

The size distribution of the various NPs was detected by a Zetasizer Nano ZS (Malvern, U.K.). TEM (JEM1400PLUS, Japan) and SEM (NovaNanoSEM430, USA) were used to observe particle size and morphology of the NPs. Western blotting analysis was performed to identify the protein profiles of EM and EhCv/siRNA NPs. Briefly, equal amounts of the total proteins from different samples quantified with BCA Protein Assay Kit were added to the 10% SDS-polyacrylamide gel to separate different molecular weights of proteins. Subsequently, the gel was stained with Coomassie brilliant blue and imaged. For western blotting assay, the proteins on the gel were then transferred to polyvinylidene fluoride (PVDF) membrane, then the membrane was blocked with 5% skimmed milk and incubated with the primary antibodies of anti-GRP94, anti-UNC93B, and anti-VAMP3. The secondary anti-goat IgG antibody was incubated with corresponding primary antibodies before imaging using an ECL Western Blotting Substrate.

The integrative hybrid nanostructure of EhCv/siRNA NPs was further determined by the colocalization of EM, Cv, and siRNA. Briefly, Cv was composed of rhodamine-labeled large single chamber vehicles (DOTAP/DOPE/Cholesterol/rhodamine-labeled DOPE = 1/1/1/0.05, mol/mol), and siRNA was labeled with Cy5, and EM was marked by antibody (primary antibodies of anti-KDEL and Alexa Fluor^@^488-labeled secondary anti-goat IgG antibody). Then the three-fluorescence dye-labeled EhCv/siRNA NPs (Cy5-siRNA:1 μM, N/P = 5/1, and EM/Cv NPs = 0.5/1 (w/w)) was assembled. Four microliters EhCv/siRNA NPs (three-fluorescence dye-labeled) were dropped on the slide and the fluorescence images were obtained using a Leica SP8 CLSM, Wetzlar, Germany) at different excitation wavelengths (Rhodamine: 561 nm, Alexa Fluor^@^488: 488 nm, Cy5: 650 nm).

Furthermore, Cv/siRNA NPs composed of Cv and Cy5-siRNA was constructed with EM marked by antibody (Alexa Fluor^@^568-labeled antibody of anti-KDEL, antibody/EM = 1/8, w/w) to obtain another two-fluorescence dye-labeled EhCv/siRNA NPs. And then MCF-7 cells (2 × 10^5^ cells per well in confocal dishes) incubated with EhCv/siRNA NPs (two-fluorescence-labeled, Cy5-siRNA:100 nM) for 4 h were observed by Leica SP8 CLSM to verify the integrative nanostructure of EhCv/siRNA NPs.

### Stability and safety of EhCv/siRNA NPs

*Hemolysis and hemagglutination test*: Erythrocytes (RBC) were isolated from Sprague-Dawley rat blood by centrifugation at 1500 × *g* for 10 min at 4 °C and were washed with physiological saline solution three times. The cell pellets were re-suspended into a 2% (v/v) erythrocyte suspension with pre-chilled PBS (0.1 M, pH = 7.38) and then added 100 μL into each well of the 96-well plates. Aliquot 100 μL of the NPs (siRNA: 200 nM, N/P = 5/1 and EM/Cv or CM/Cv = 0.5/1 (w/w)) was into each well of the plates. 5% glucose and Triton X-100 PBS (1%, v/v) were used as the negative and positive control, respectively. After 1 h incubation at 37 °C, each sample was divided into two EP tubes. One tube was used for hemagglutination test. Briefly, 10 μL samples were dropped on the slide and the images were dehydrated in a microscope (Wetzlar, Germany). Another tube was centrifuged and the supernatant was transferred to a new 96-well plate, and the image was captured by camera (Nikon D5100, Japan) and the absorbance was measured at 540 nm by the multiskan Mk3 microplate reader (Thermo scientific, USA). And the relative hemolysis rate was calculated by the formula as following: ([Abs]sample − [Abs]buffer)/([Abs]Triton X-100 − [Abs]buffer) × 100%.

### Serum stability assay

The mixture of 100 μL of serum and 100 μL of different NPs (siRNA 200 nM, N/P = 5/1 and EM/Cv or CM/Cv = 0.5/1(w/w)) was added into each well of the 96-well plates. 5% glucose was used as a negative control and 0.1% Triton X-100 PBS (1%, v/v) or PEI_25k_/siRNA (siRNA 200 nM, N/P = 5/1) was used as the positive control. At predetermined time points, the absorbance of each sample was measured at 630 nm by the multiskan Mk3 microplate reader (Thermo scientific, USA).

### Gel retardation assay

To investigate the protective effects of different NPs on siRNA, the gel retardation assay was conducted. These NPs (siRNA: 2 μM) were mixed with FBS at the volume ratio of 1:1 and incubated for different time points at 37 °C. Free siRNA was used as the control. The samples were kept in −20 °C before gel retardation assay. After that, the samples were tackled with 6× loading buffer containing 3% SDS on a 2% agarose gel containing 0.5 mg mL^−1^ Exred. Electrophoresis was performed at 80 mV for 3 min, subsequently at 100 mV for 15 min, and these resulting gels were photographed under UV illumination.

### In vitro cytotoxicity assay

In vitro cytotoxicity of NPs on MCF-7 cells was performed by MTT assay. Briefly, MCF-7 cells were seeded in 96-well plates at a density of 10,000 cells per well for 24 h proliferation. The cells were treated with 200 μL Opti-MEM containing various NPs (the final concentration of siNC 100 nM) for 6 h incubation. After that, the culture medium was refreshed by RPMI-1640 medium containing 10% FBS and the cells were continuously incubated for 24 h. Then 20 μL MTT (5 mg mL^−1^) solution was dropped into each well and incubated for another 4 h. Then the culture medium was removed, and the formazan crystals were dissolved in 100 μL dimethyl sulfoxide, and the absorbance values were detected by a microplate reader (Multiskan Mk3, Thermo scientific, US) at the wavelength of 490 nm. Cell viability (%) = [OD_490_ (sample)/OD_490_ (control)] × 100%.

### Evaluation of cellular uptake

The cellular uptake of EhCv/siRNA NPs in different cells was further investigated. MCF-7, HT-1080, HepG2 cells, and A549 cells were seeded 2.5 × 10^5^ per well in 6-well plates. After 24 h proliferation, the Cv/siRNA NPs, EhCv/siRNA NPs and ChCv/siRNA NPs (EM and CM extracted from MCF-7 cells or HT-1080 cells) containing FAM-labeled siRNA at the final concentration of 100 nM were exposed to cells and incubated for an additional 6 h at 37 °C. After incubation, the cells were harvested and washed three times with pre-cooled PBS solution, and intracellular fluorescence intensities were detected by a FACS Calibur flow cytometry (Becton Dickinson, San Jose, CA, USA) immediately.

In addition, the MCF-7 and HT-1080 cells (2 × 10^5^ cells per well in confocal dishes) were inducted with the Cv/siRNA NPs, EhCv/siRNA NPs, and ChCv/siRNA NPs (EM and CM extracted from MCF-7 cells and HT-1080 cells) NPs containing Cy5-labeled siRNA at the final concentration of 100 nM for an additional 6 h at 37 °C, respectively. After incubation, the cells were harvested and washed three times with pre-cooled PBS solution, followed by fixation with 4% paraformaldehyde for 15 min and punched by Triton X-100 PBS (0.1%, v/v) for 1 min. Then the nucleus and cell membrane were stained by Hoechst 33258 (5 μg mL^−1^ for 10 min) and rhodamine phalloidin (1.5 U mL^−1^ for 10 min), respectively. Finally, the intracellular fluorescence distribution was visualized under a Leica SP8 CLSM (Wetzlar, Germany).

### Investigation of endocytosis pathways

MCF-7 cells were plated in 12-well tissue culture plates at 2 × 10^5^ cells per well 24 h prior to transfection. After the cells were pretreated with different inhibitors (amiloride 0.5 mM; chlorpromazine 10 μg mL^−1^; β-CD 5 mM; genistein 40 μg mL^−1^; nystatin 40 μg mL^−1^), the culture medium was replaced with Opti-MEM before transfection. Then three NPs (FAM-labeled siRNA :100 nM) were exposed to cells and incubated for an additional 4 h at 37 °C. During incubation, the concentration of inhibitors or temperature was consistent with the pre-treatment. After incubation, the cells were harvested and washed three times with pre-cooled PBS solution and the uptake of FAM-labeled siRNA was detected by the FACS Calibur flow cytometry (Becton Dickinson, San Jose, CA, USA) at the wavelength of 488 nm immediately.

Confocal microscopy was also used to observe the internalization routes of the NPs. MCF-7 cells were plated in confocal dishes (15 mm) at 2 × 10^5^ cells per well 24 h prior to transfection. Then various NPs containing Cy5-labeled siRNA at the final concentration of 100 nM were exposed to cells and incubated for additional 4 h at 37 °C. Before the end of incubation, TRF (Alexa Fluor 488-conjugated Transferrin, 10 μg mL^−1^, incubated for 30 min), CT-B (Alexa Fluor 488 Conjugated Cholera Toxin Subunit B, 2 μg mL^−1^, incubated for 30 min), and Dextran (FITC Conjugates, 1 mg mL^−1^, incubated for 1 h) were added into the incubation system, respectively. After transfected, the cells were washed three times with PBS followed by fixation with 4% paraformaldehyde for 15 min, then the nucleus were stained by Hoechst 33342 (5 μg mL^−1^ for 10 min). Cells were visualized under a Leica SP8 CLSM (Wetzlar, Germany).

### In vitro gene silencing effects

To detect the relative expression level of EGFR mRNA, the cells treated with different NPs were analyzed by reverse transcriptase-PCR (RT-PCR) assay. First, MCF-7 cells (2 × 10^5^ cells per well) were seeded into 12-well tissue culture plates. After 24 h proliferation, various NPs containing siEGFR (0, 5, 10, 30, 50, 70, 100, and 200 nM) were exposed to cells and incubated for an additional 6 h at 37 °C. After refreshed cell culture medium, the cells were incubated for another 24 h of proliferation. Total RNA was extracted using TRIZOL® reagent, then reverse transcribed with GoScript™ Reverse Transcription System (A5001) (Promega, USA). PCR was performed on cDNA pre-treated with GoTaq®qPCR Master Mix (A6002) (Promega, USA) on Real-Time PCR amplifier (MX3005P, Stratagene, USA). All quantitation was normalized to an endogenous control glyceraldehyde 3-phosphate dehydrogenase (GAPDH). The relative quantitation value of each target gene is expressed as 2^−(Ct−Cc)^ (C_t_ and C_c_ represent the mean threshold cycle differences of treatment and control after normalizing to GAPDH, respectively). In addition, the relative expression level of EGFR mRNA on HT-1080 cells was also detected RT-PCR assay.

For western blotting analysis of EGFR protein expression level, similar cell culture and treatments were performed as described above. After 48 h incubation, the cells were washed with pre-cooled PBS and then re-suspended in 100 μL RIPA lysis buffer supplemented with 1% proteinase inhibitor cocktail. The cell lysates were incubated on ice for 30 min and vortexed every 10 min. The total protein was gathered by centrifugation at 12,000 × *g* for 1 h and the concentration was determined with the BCA Protein Assay Kit. Total protein (30 μg) was loaded on 12% SDS- polyacrylamide gel and electrophoresed at 80 mV for 30 min and 120 mV for 2 h. Then the proteins were transferred to PVDF membranes at 200 mA for 120 min and then blocked with 5% skimmed milk on a horizontal shaker for 2 h. The membranes were incubated with the 1:500 EGFR monoclonal antibody (Santa Cruz Biotechnology) overnight at 4 °C followed by incubation with horseradish peroxidase-conjugated goat anti-rabbit antibodies (1:2000 Zhongshan Goldenbridge Biotechnology Co. Ltd., Beijing, China) at room temperature for 2 h. Finally, the membranes were exposed using a Bio-rad ChemiDoc XRS System. β-Actin was used as an endogenous control.

### Assay of intracellular trafficking pathway

MCF-7 cells (2 × 10^5^ cells per well) were seeded into confocal dishes. After 24 h incubation, the NPs containing Cy5-labeled siRNA (100 nM) in Opti-MEM were added into each dish for different time periods (2, 4, 6 h). For the distribution in Golgi, Golgi-Tracker (5 μM) and Hoechst 33342 (5 μg mL^−1^) were added in cell culture medium and incubated for 30 min at 37 °C to label Golgi and nucleus at the end of transfection, respectively. Afterwards, the cells were washed with PBS for three times followed by fixation with 4% paraformaldehyde for 15 min. For the distribution in ER and lysosomes, Hoechst 33342 (5 μg mL^−1^) was added in cell culture medium and incubated for 30 min at 37 °C to label nucleus at the end of transfection. Afterwards, the cells were washed with PBS for three times followed by fixation with 4% paraformaldehyde for 15 min and punched by Triton X-100 PBS (0.1%, v/v) for 5 min, then blocked with 2% bovine serum albumin in Tween-PBS (0.1%, v/v) for 1 h. The cells were then incubated overnight at 4 °C with Anti-KDEL and Anti-M6PR (Alexa Fluor 568 labeled, 2.5 μg mL^−1^) to label ER and lysosomes, respectively. Next, the cells were washed with PBS for three times. Intracellular distribution of Cy5-labeled siRNA was observed with a Leica SP8 CLSM.

To certify the function of COPI vesicles in the transport pathway of EhCv/siRNA NPs, the distribution in ER and Golgi with or without GCA (a powerful Golgi inhibitor, specificity against the assembly of COPI coated vesicles) were detected by Leica SP8 CLSM. Briefly, the MCF-7 cells (2 × 10^5^ cells per well in confocal dishes) were incubated with EhCv/siRNA NPs for 4 h with or without GCA (10 μM), followed by treatment described as above and the cellular uptake intensities of the EhCv/siRNA NPs (+GCA, 10 μM) were detected by the FACS Calibur flow cytometry as the procedure described above. Meanwhile, the 24-h gene silencing effect on EGFR mRNA after incubation with EhCv/siEGFR NPs (+GCA, 10 μM) also was examined by RT-PCR.

### Functional verification of protein-removed EM

To validate protein function, the proteins in EM were removed by sodium carbonate.^37^ Briefly, the extracted EM was diluted 100-fold with 100 mM sodium carbonate at pH 11.5, and incubated at 4 °C for 30 min. The suspensions were centrifuged at 4 °C for 1 h at 10,000 × *g* in polycarbonate tubes in the Beckman 50 Ti rotor. The supernatants were decanted and the membrane pellets were gently rinsed once with ice-cold distilled water. EM that proteins had been removed was named as rEM. The western-blotting analysis was performed to identify the protein components of rEM and rEhCv/siRNA NPs as the method described before. Furthermore, rEM was used to construct with Cv/siRNA NPs for hybrid rEhCv/siRNA NPs. The cellular uptake intensities and the distribution of the rEhCv/siRNA NPs in organelles were detected by the FACS Calibur flow cytometry and CLSM as described by the procedure above. Meanwhile, the 24-h gene silencing effect on EGFR mRNA after incubation with rEhCv/siEGFR NPs also was examined by RT-PCR.

To further confirm the key role of ER-resident proteins on transporting siRNA-loaded nanoplexes, its inherent KDEL signal peptide was used as a ligand first to be conjugated with HA by amidation reaction and the hyaluronan-KDEL polymer (HK) was obtained. The HK@Cv/siRNA and HA@Cv/siRNA NPs were prepared by coating the resulting HK polymer and HA polymer on the Cv/siRNA NPs with electrostatic interaction, respectively. The cellular uptake, gene silencing effects, and intracellular fluorescence colocalization with organelles of HK@Cv/siRNA NPs were characterized and evaluated in in vitro cell experiments.

### Preparation and characterization of HK polymers and HK@Cv/siRNA NPs

First, hyaluronan-KDEL graft polymers (HK) were synthesized. Briefly, HA (12.5 kDa, 1 g, 2.5 mmol) was dissolved in 100 mL of degassed deionized water. 1-(3-Dimethylaminopropyl)-3-ethylcarbodiimide hydrochloride (EDC, 959 mg, 3.75 mmol) and *N*-hydroxysuccinimide (NHS, 575 mg, 3.75 mmol) were slowly added to the HA solution. After 20 min stirring, KDEL peptides (500 mg) was added to the mixture. The reaction solution was adjusted at pH 4–5 and kept stirring for 2 days at room temperature. After the end of reaction, the product was purified by dialysis (MWCO = 8000 Da) against distilled water and lyophilized. The structures of all products were confirmed by ^1^H-NMR (AVANCE III, 400 MHz, Bruker, Billerica, MA) spectrometry.

Afterwards, 25 μL 5% glucose solution or PBS of siRNA (4 μM) was mixed with Cv to construct Cv/siRNA NPs (N/P = 5/1) in the same volume, then allowed to incubate for 15 min at 37 °C. Subsequently, 50 μL Cv/siRNA NPs was dropped into the same volume of HA or HK solution at different C/N ratios, followed by 15 min incubation at 37 °C, then the HA@Cv/siRNA NPs or HK@Cv/siRNA NPs were prepared, respectively. For optimizing the formulation of two NPs, the size distribution with various C/N ratios of HA or HK and Cv was detected by a Zetasizer Nano ZS (Malvern, U.K.). Moreover, to investigate the siRNA-loading capability at different C/N ratios, the gel retardation assay was conducted as before.

### Stability and safety of HK@Cv/siRNA NPs

*Hemolysis and hemagglutination test*: Erythrocytes (RBC) were isolated from Sprague-Dawley rat blood by centrifugation at 1500 × *g* for 10 min at 4 °C and were washed with physiological saline solution three times. The cell pellets were re-suspended into a 2% (v/v) erythrocyte suspension with pre-chilled PBS (0.1 M, pH = 7.38) and then 100 μL was added into each well of the 96-well plates. Aliquot 100 μL of the NPs (siRNA: 200 nM, N/P = 5/1, and C/N ratio = 2/1) was added into each well of the plates. 5% glucose and Triton X-100 PBS (1%, v/v) were used as the negative and positive control, respectively. After 1 h incubation at 37 °C, each sample was divided into two EP tubes. One tube was used for hemagglutination test. Briefly, 10 μL samples were dropped on the slide and the images were dehydrated in a microscope (Wetzlar, Germany). Another tube was centrifuged and the supernatant was transferred to a new 96-well plate, and the image was captured by camera (Nikon D5100, Japan) and the absorbance was measured at 540 nm by the multiskan Mk3 microplate reader (Thermo Scientific, USA). And the relative hemolysis rate was calculated by the formula as following: ([Abs]sample − [Abs]buffer)/([Abs]Triton X-100 − [Abs]buffer) × 100%.

### Gel retardation assay

The mixture of 100 μL of serum and 100 μL of different NPs (siRNA 200 nM, N/P = 5/1, and C/N ratio = 2/1) was added into each well of the 96-well plates. 5% glucose was used as a negative control and 0.1% Triton X-100 PBS (1%, v/v) or PEI_25k_/siRNA (siRNA 200 nM, N/P = 5/1) was used as the positive control. At predetermined time points, the absorbance of each sample was measured at 630 nm by the multiskan Mk3 microplate reader (Thermo scientific, USA).

### Cellular uptake of HK@Cv/siRNA NPs

The cellular uptake of HK@Cv/siRNA NPs in A549 cells was further investigated. A549 cells were seeded at 2.5 × 10^5^ per well in 6-well plates. After 24 h proliferation, the Cv/siRNA NPs, HA@Cv/siRNA NPs, and HK@Cv/siRNA NPs containing FAM-labeled siRNA at the final concentration of 100 nM were exposed to cells and incubated for an additional 6 h at 37 °C. After incubation, the cells were harvested and washed three times with pre-cooled PBS solution, and intracellular fluorescence intensities were detected by a FACS Calibur flow cytometry (Becton Dickinson, San Jose, CA, USA) immediately.

In addition, the A549 cells (2 × 10^5^ cells per well in confocal dishes) were inducted with the Cv/siRNA NPs, HA@Cv/siRNA NPs, and HK@Cv/siRNA NPs containing Cy5-labeled siRNA at the final concentration of 100 nM for an additional 6 h at 37 °C, respectively. After incubation, the cells were harvested and washed three times with pre-cooled PBS solution, followed by fixation with 4% paraformaldehyde for 15 min and punched by Triton X-100 PBS (0.1%, v/v) for 1 min. Then the nucleus and cell membrane were stained by Hoechst 33258 (5 μg mL^−1^ for 10 min) and rhodamine phalloidin (1.5 U mL^−1^ for 10 min), respectively. Finally, the intracellular fluorescence distribution was visualized under a Leica SP8 CLSM (Wetzlar, Germany).

### In vivo antitumor effects

In situ MCF-7 tumor models were prepared by local injection of 2 × 10^6^ cells under the right pads of female BALB/c nude mice. Then BALB/c nude mice were randomly divided into six groups (*n* = 6) and injected peritumorally with PBS, EhCv/siEGFR NPs, ChCv/siEGFR NPs, Cv/siEGFR NPs, rEhCv/siEGFR NPs, and EhCv/siNC NPs at a siRNA dose (0.5 mg kg^−1^) in 150 μL of PBS buffer once every 2 days. All the mice were administered five consecutive injections, the tumor growth was monitored by measuring perpendicular diameters using a calliper, and tumor volume was calculated as follows: *V* = *L* × *W*^2^/2, where *W* and *L* represent the shortest and longest diameters, respectively. Body weight and tumor size of each mouse were measured daily. The day before the first dose was defined as day 0. Three days after the last injection, the animals were sacrificed to harvest tumors and the image was captured by camera (Nikon D5100, Japan) and weighted. At 0 and 12th day after the beginning of treatment, the measurement of HGB was tested. Then the EGFR protein in tumor tissues were determined by western blot assay and IHC. Meanwhile, the pathological changes of other organs, including heart, liver, spleen, lung, and kidney, were analyzed by hematoxylin–eosin staining.

All animal testing and research were complied with the relevant ethical regulations. All animal procedures were approved by the Peking university experimental animal welfare ethics review committee.

### Statistical analysis

The results were expressed as mean ± standard deviation. For statistical analysis between two groups, Student’s *t* test for independent means was applied. Comparisons between multiple groups were made by one-way ANOVA followed by least significant difference multiple comparison test. Statistical analysis was performed using the SPSS 16.0 software (SPSS Inc., Chicago). A value of *p* < 0.05 was considered as statistically significant.

### Reporting summary

Further information on research design is available in the Nature Research Reporting Summary linked to this article.

## Supplementary information

Supplementary Information

Reporting Summary

Source Data

## Data Availability

Source data are provided as a Source Data file. The Source Data file are available from 10.6084/m9.figshare.8081408. All Source Data are available within the article and its Supplementary Information files or from the corresponding authors upon reasonable request. A reporting summary for this article is available as a Supplementary Information file.
