# Supplementary material for: Regulating intracellular fate of siRNA by endoplasmic reticulum membrane-decorated hybrid nanoplexes
Source: Nat Commun. 2019 Jun 20;10:2702. doi: 10.1038/s41467-019-10562-w (PMC6586638; doi:10.1038/s41467-019-10562-w)
Supplement: Supplementary file 1 — Supplementary Information [file 41467_2019_10562_MOESM1_ESM.pdf]

Supplementary Information

Regulating Intracellular Fate of siRNA by Endoplasmic Reticulum Membrane-Decorated Hybrid Nanoplexes

Qiu *et al.*

Supplementary Results and Discussion

Optimization of EhCv/siRNA NPs

The ER membranes, extracted according to the operation of the calcium chloride precipitation and gradient centrifugation,[1, 2] presented a reddish-brown color (**Supplementary Fig.1B**). Cationic vehicles (Cv) were assembled with DOTAP, DOPE and cholesterol by the film dispersion method. Since it could effectively load siRNA when the N/P ratio was above 2/1, the Cv/siRNA NPs (N/P=5/1) were selected as the typical formulation for the assembly of EhCv/siRNA NPs (**Supplementary Fig.1A**). The hydrodynamic sizes, zeta potential, the capability of siRNA loaded and cellular uptake in different weight ratios were detected for the prescription optimization of EhCv/siRNA NPs (**Supplementary Fig.1C**, **Supplementary Fig.1D** and **Supplementary Fig.1E**). And EhCv/siRNA NPs (N/P = 5/1, EM/Cv = 0.5/1(w/w)) were selected for the subsequent experimental study. The ChCv/siRNA NPs were prepared by replacing EM with cancer cell membrane (CM) (**Supplementary Fig.1F** and **1G**).

Stability and Safety of EhCv/siRNA NPs

The haemolysis assay was used to evaluate the biosafety of EhCv/siRNA NPs. As shown in **Supplementary Fig.3**, EhCv/siRNA NPs presented a serum stability and hemagglutination effect like Cv/siRNA NPs and 5% glucose, however, the average cell viability of EhCv/siRNA NPs on MCF-7 cells (above 90%) was higher than that of Cv/siRNA NPs (~ 77%). Meanwhile, the haemolysis rate of EhCv/siRNA NPs (~ 2.5%) was lower than that of Cv/siRNA NPs (~ 13%) significantly. Those results meant that EhCv/siRNA NPs had a better safety than Cv/siRNA NPs due to its lower surface charge and biocompatibility derived from EM. Besides, the results from gel retardation assay (**Supplementary Fig.3F**) confirmed that siRNAs of three NPs could be protected for 36 h after being tackled with FBS. And they presented similar degree of degradation because siRNAs were absorbed on the outer layer of hybrid liposomes, which further certified that the EM or CM was rearranged into the lipid layer instead of coating outer.

Internalization Pathway of EhCv/siRNA NPs

Generally, there are three pathways involved in the internalization of nanoparticles, including clathrin-mediated endocytosis (CME), caveolae-mediated endocytosis (CeME) and micropinocytosis.[3] In this study, both approaches of pathway inhibitors and channel markers were applied to gain insight into the internalization pathways of various NPs on MCF-7 cells, followed by the quantitative analysis of FCM assay and the qualitative analysis of CLSM

observation, respectively. Four inhibitors were used to block different pathways, including CME (40 μg mL^{-1} nystatin, 5 μg mL^{-1} chlorpromazine), macropinocytosis (0.25 mM amiloride) and CeME (50 μg mL^{-1} genistein, 5 mM β-CD). Meanwhile, three kinds of known fluorescent-labeled channel markers, Alexa 488-Tf (CME), Alexa 488-CTB (CeME) and FITC-dextran (macropinocytosis), were used to trace the internalization pathways. As shown in **Supplementary Fig.7A** and **Supplementary Fig.7B**, various inhibitors could obviously inhibit the cellular uptake of EhCv/siRNA NPs with a different degree, which implied that they might entry into cancer cells by the mix pathway. This probable mechanism was further proved by the strong colocalization (yellow) between Cy5-labeled siRNA (red) and three channel markers (green). Similarly, although amiloride didn't inhibit the cellular uptake of ChCv/siRNA NPs and chlorpromazine didn't decrease the cellular uptake of Cv/siRNA NPs, there still were obvious colocalization dots of ChCv/siRNA NPs and FITC-dextran or Cv/siRNA NPs and Alexa Fluor 488-Transferrin, indicating that three pathways were involved in the internalization of ChCv/siRNA NPs and Cv/siRNA NPs. Moreover, the sum of colocalization rate of all markers was set as 100% to calculate the proportional value of three main uptake channels (**Supplementary Fig.7C**). Three NPs had a similar channel ratio of micropinocytosis, but another two channels ratio was different, including CeME (EhCv/siRNA NPs > ChCv/siRNA NPs > Cv/siRNA NPs) and CME (Cv/siRNA NPs > ChCv/siRNA NPs > EhCv/siRNA NPs). As reported previously, some researchers believed that those small molecules endocytosed by CeME could bypass the endosome-lysosomal pathway and directly transported to the Golgi apparatus or ER.[4, 5] Therefore, the higher ratio of CeME in the endocytosis pathway implied that the EhCv/siRNA NPs might transported to the Golgi apparatus or ER directly, and thereby avoid the lysosomal degradation of siRNA.

Preparation and Characterization of HK polymers and HK@Cv/siRNA NPs

The hyaluronan-KDEL polymer (HK) was successfully synthesized by conjugating the amine group of KDEL with carboxyl group of hyaluronic acid via EDC/NHS coupling technique (**Supplementary Fig.10A**). The structures of HK polymers were confirmed by 1H NMR analysis (**Supplementary Fig.10B**). The proton peak of HA appeared between 1.8~2.0 ppm (a, single) and 3.0~4.0 ppm (multiple), where the peak between 1.8~2.0 ppm (b) was representative CH3-group of KDEL. To prepare optimal HK@Cv/siRNA and HA@Cv/siRNA NPs, the Cv/siRNA NPs (N/P ratio of 5/1) were combined with negative HK by electrostatic interaction at different molar ratios of C/N (C: carboxyl group in HK or HA, N: amidogen in DOTAP) (**Supplementary Fig.10B**). After HK decoration (C/N=2/1), HK@Cv/siRNA NPs

obtained the slightly bigger hydrodynamic diameter (184.1 ± 4.9 nm) and obvious lower zeta-potentials (-29.9 ± 1.7 mV) like HA@Cv/siRNA NPs (190.0 ± 3.8 nm, -27.0 ± 0.3 mV) (**Supplementary Fig.10C~F** and **Supplementary Tab.1**), which were resulted from the decoration of negative-charged HK or HA.

The hemagglutination assay, haemolysis assay and gel retardation assay were performed to evaluate the safety and stability of HK@Cv/siRNA NPs, respectively. In **Supplementary Fig.11A**, compared to PEI_{25k}/siRNA, HK@Cv/siRNA NPs exhibited a negilable hemagglutination effect. The haemolysis effects of HK@Cv/siRNA NPs on erythrocyte (~ 2%) was significantly lower than that of Cv/siRNA NPs (~ 13%) ($p < 0.01$, t-test) (**Supplementary Fig.11B**). Besides, the results of serum protective effects (**Supplementary Fig.11C**) indicated that HK@Cv/siRNA NPs could protect siRNA against RNAase in FBS for 36 h, which was longer than Cv/siRNA (24 h). Above all, these results demonstrated that HK@Cv/siRNA NPs had a better safety and stability for *in vitro* and *in vivo* potential application.

Supplementary References

1. Chanat, E., Le Parc, A., Lahouassa, H. & Badaoui, B. Isolation of Endoplasmic Reticulum Fractions from Mammary Epithelial Tissue. *J. Mammary Gland Biol. Neoplasia* **21**, 1-8 (2016).
2. Plonne, D. et al. Separation of the intracellular secretory compartment of rat liver and isolated rat hepatocytes in a single step using self-generating gradients of iodixanol. *Anal. Biochem.* **276**, 88-96 (1999).
3. Vercauteren, D. et al. On the cellular processing of non-viral nanomedicines for nucleic acid delivery: mechanisms and methods. *J. Control. Release* **161**, 566-581 (2012).
4. Reilly, M.J., Larsen, J.D. & Sullivan, M.O. Polyplexes Traffic through Caveolae to the Golgi and Endoplasmic Reticulum en Route to the Nucleus. *Mol. Pharmaceut.* **9**, 1280-1290 (2012).
5. Kim, A.J., Boylan, N.J., Suk, J.S., Lai, S.K. & Hanes, J. Non-degradative intracellular trafficking of highly compacted polymeric DNA nanoparticles. *J. Control. Release* **158**, 102-107 (2012).

Supplementary Table 1. Size, polydispersity index (PDI) and zeta potential of nanoplexes (mean ± SD, n = 3).

Nanoplexes	Particle size (d, nm)	Polydispersity Index (PDI)	Zeta potential (mV)
EM	363.2±35.3	0.33±0.03	-21.2±0.6
CM	208.1±6.1	0.25±0.01	-27.0±0.9
rEM	297.2±35.4	0.24±0.02	-24.2±0.3
EhCv/siRNA	188.2±2.6	0.16±0.06	29.5±0.8
ChCv/siRNA	191.4±8.3	0.25±0.01	27.9±3.8
rEhCv/siRNA	197.0±11.7	0.20±0.02	30.4±4.1
Cv/siRNA	160.4±1.9	0.16±0.01	45.1±2.7
HK@ Cv/siRNA	184.1±4.9	0.24±0.01	-29.9±1.7
HA@Cv/siRNA	190.0±3.8	0.22±0.02	-27.0±0.3

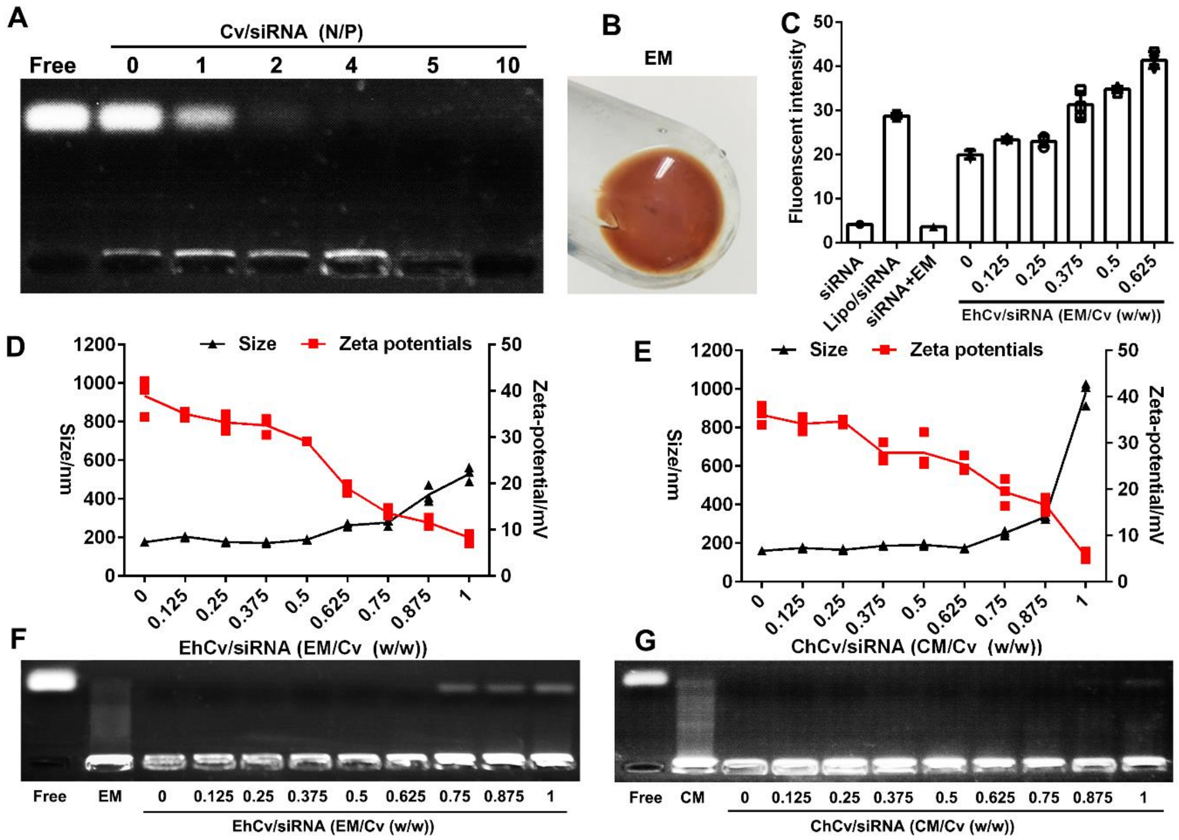

Supplementary Figure 1. Formulation optimization of EhCv/siRNA NPs and ChCv/siRNA NPs. **A**) The siRNA loading capability of Cv with different N/P detected by the gel retardation assay (siRNA: 1 μM). **B**) ER membrane extracted from MCF-7 cells. **C**) The cellular uptake capability of EhCv/siRNA NPs on MCF-7 cells. The final concentration of FAM-labled siNC was 100 nM and the data were shown as mean ± SD (n = 3). **D**) and **E**) The optimization of EhCv/siRNA NPs and ChCv/siRNA NPs based on the size and zeta potentials measured by Zetasizer Nano ZS (siRNA: 100 nM, the data were shown as mean ± SD (n = 3)).**F**) and **G**) The siRNA loading capability of EhCv/siRNA NPs and ChCv/siRNA NPs with different weight ratios detected by the gel retardation assay (siRNA: 1 μM, N/P=5/1).

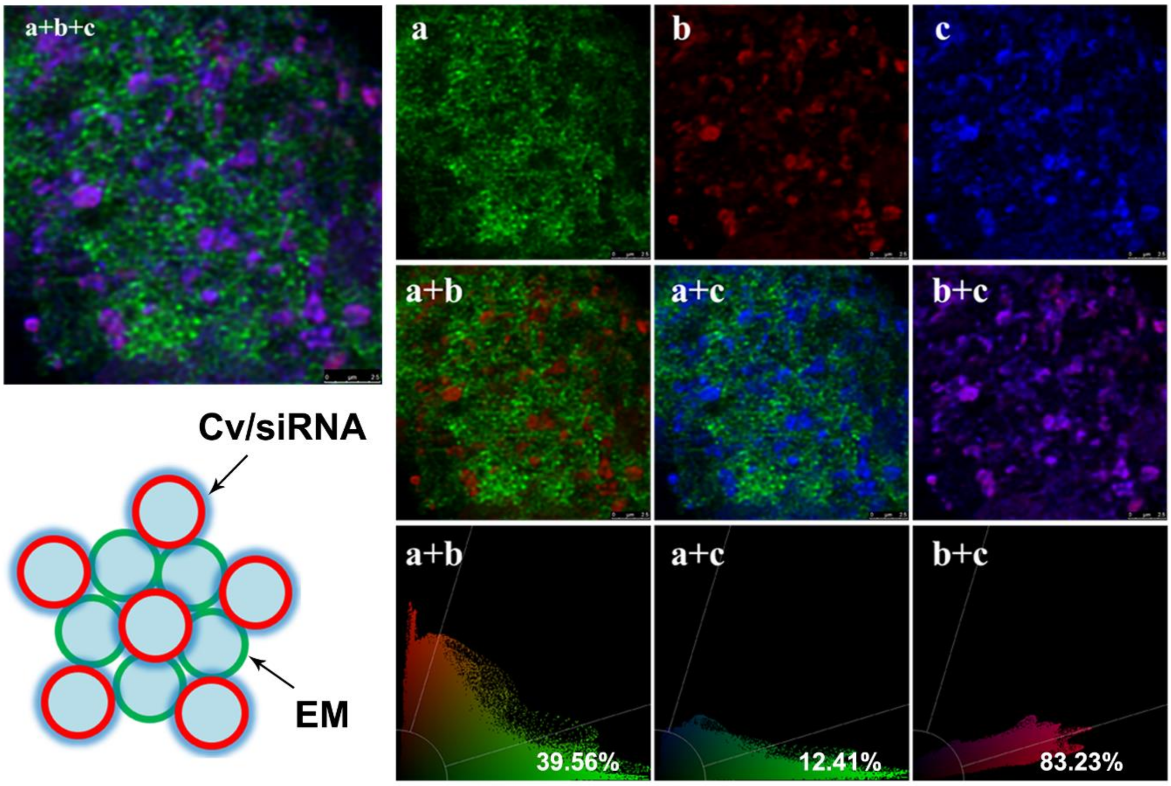

Supplementary Figure 2. The fluorescence colocalization of EM and Cv/siRNA NPs before ultrasounic treatment. EM, Cv and siRNA were labled by Alexa Fluor 488-labeled EM (a, green), rhodamine-labeled Cv (b, red) and Cy5-siRNA (c, blue), respectively. The colocalization ratio was calculated by Pearson's correlation coefficient.

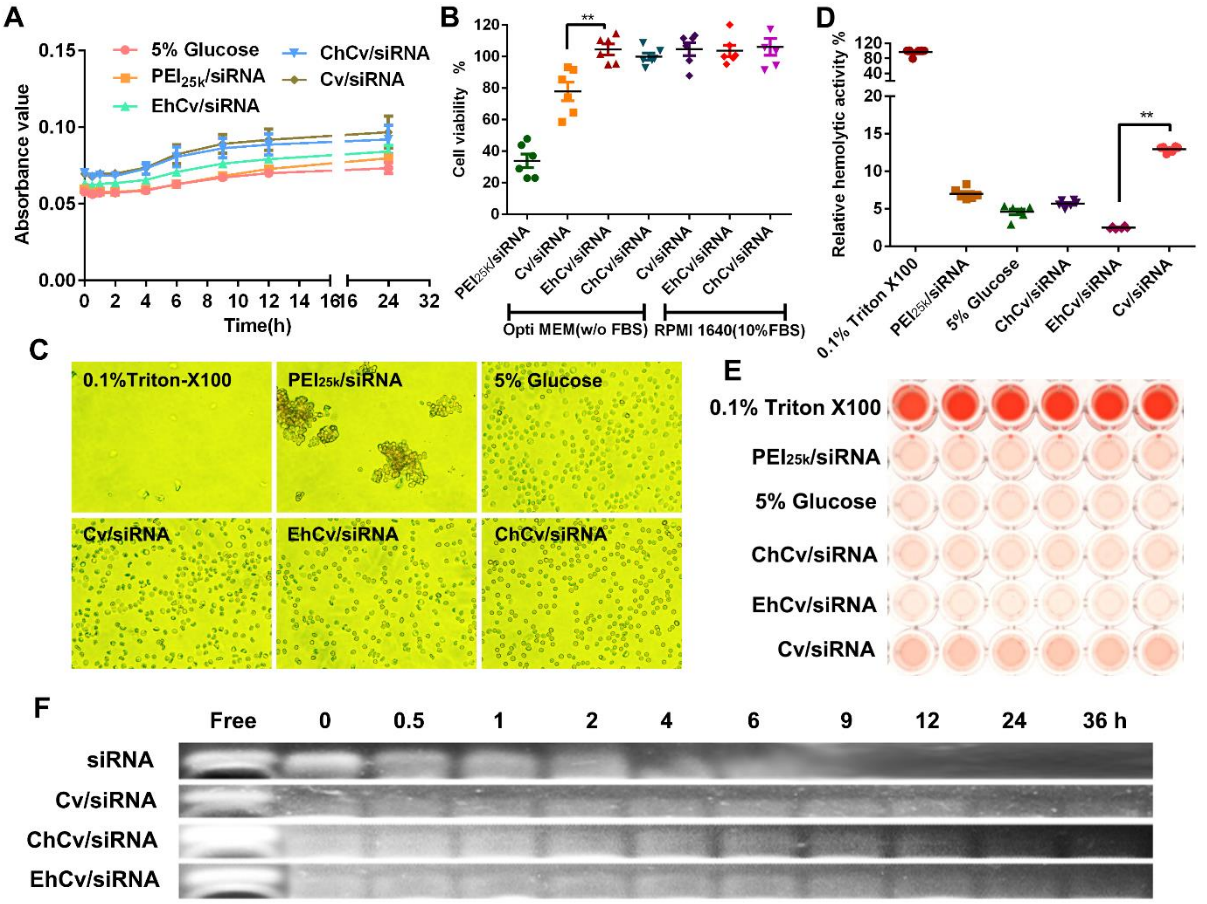

Supplementary Figure 3. Evaluation of safety and stability**. A**) The stabilities of different NPs in serum. **B**) Cytotoxicity of various NPs on MCF-7 cells. **C**) The hemagglutination test, **D**) and **E**) the hemolysis activity. **F**) The protective efficacy of various NPs on siRNA detected by gel retardation assay (siRNA: 1μM). The final concentration of siNC was 100 nM (**A ~ E**) and the data were shown as mean ± SD (n = 6), $^{**}p < 0.01$, t-test.

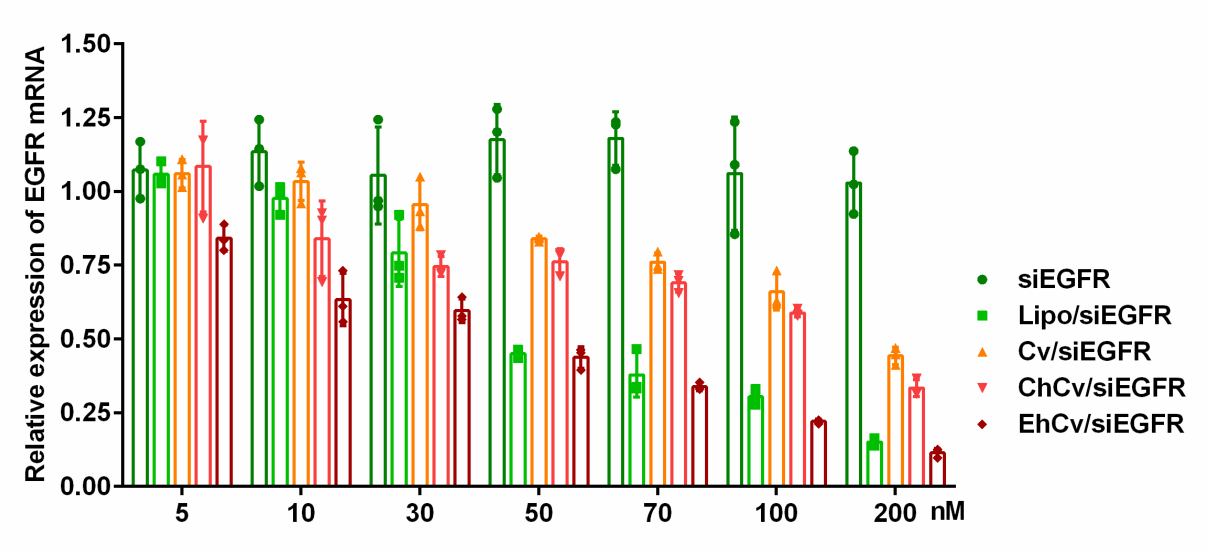

Supplementary Figure 4. Relative levels of EGFR mRNA on MCF-7 cells detected by RT-PCR. The data were shown as mean ± SD (n = 3).

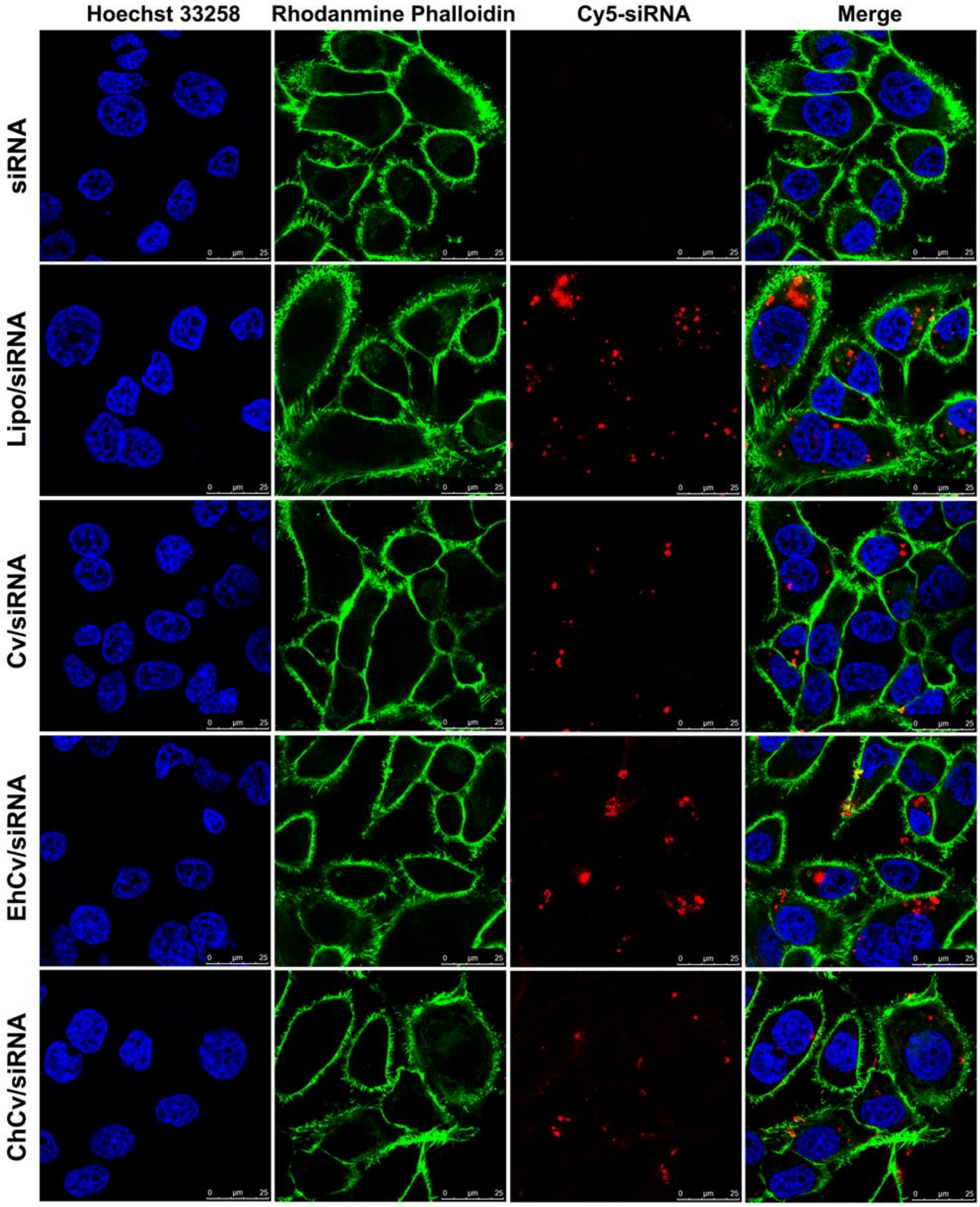

Supplementary Figure 5. Intracellular fluorescence on MCF-7 cells were detected by confocal microscopy after 6 h incubation of various NPs (Cy5-labeled siNC:100 nM, red). The nucleus and cytomembrane were respectively stained by Hoechst 33258 (blue) and rhodamine-labeled phalloidin (green).

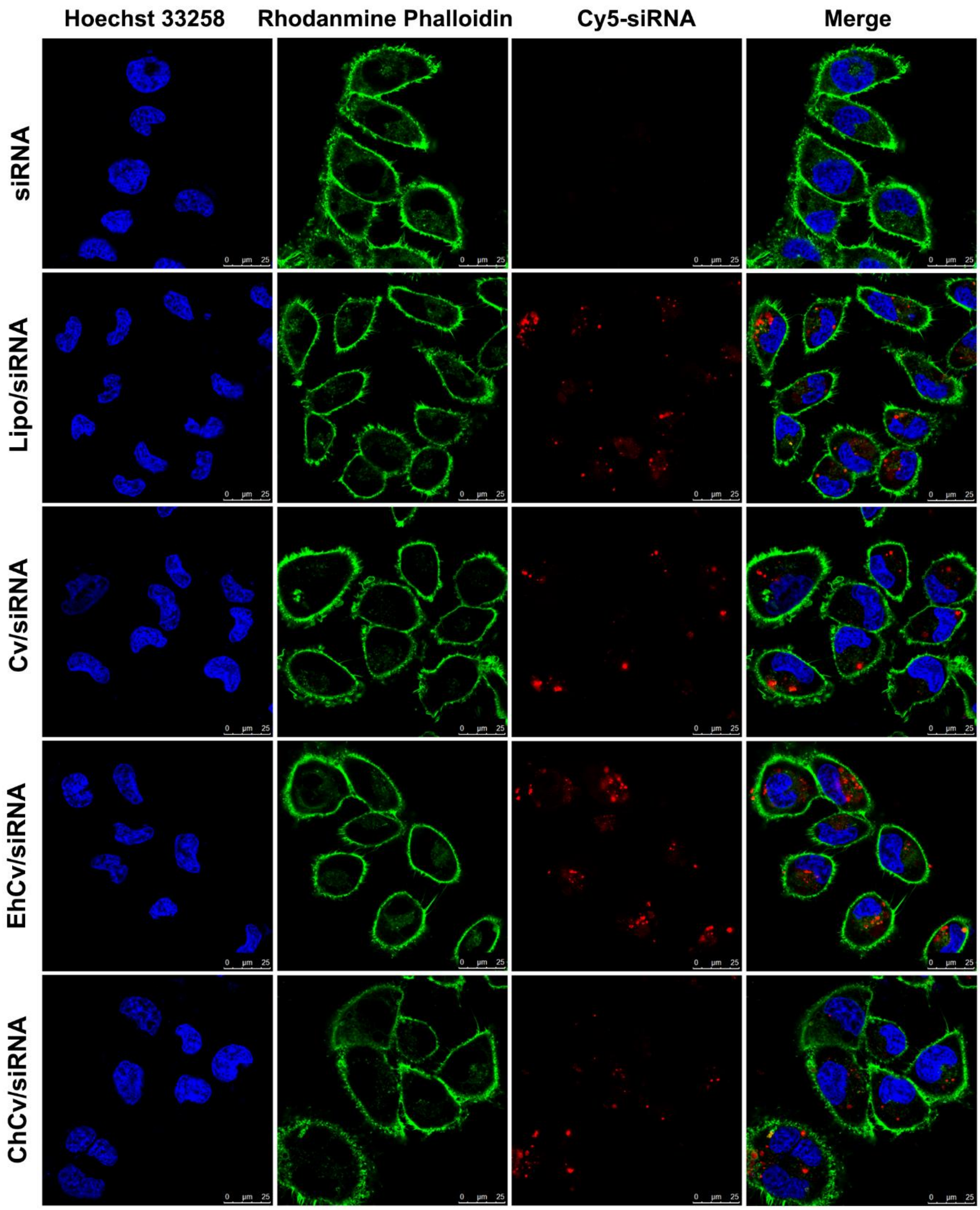

Supplementary Figure 6. Intracellular fluorescence on HT-1080 cells were detected by confocal microscopy after 6 h incubation of various NPs (Cy5-labeled siNC:100 nM, red). The nucleus and cytomembrane were respectively stained by Hoechst 33258 (blue) and rhodamine-labeled phalloidin (green).

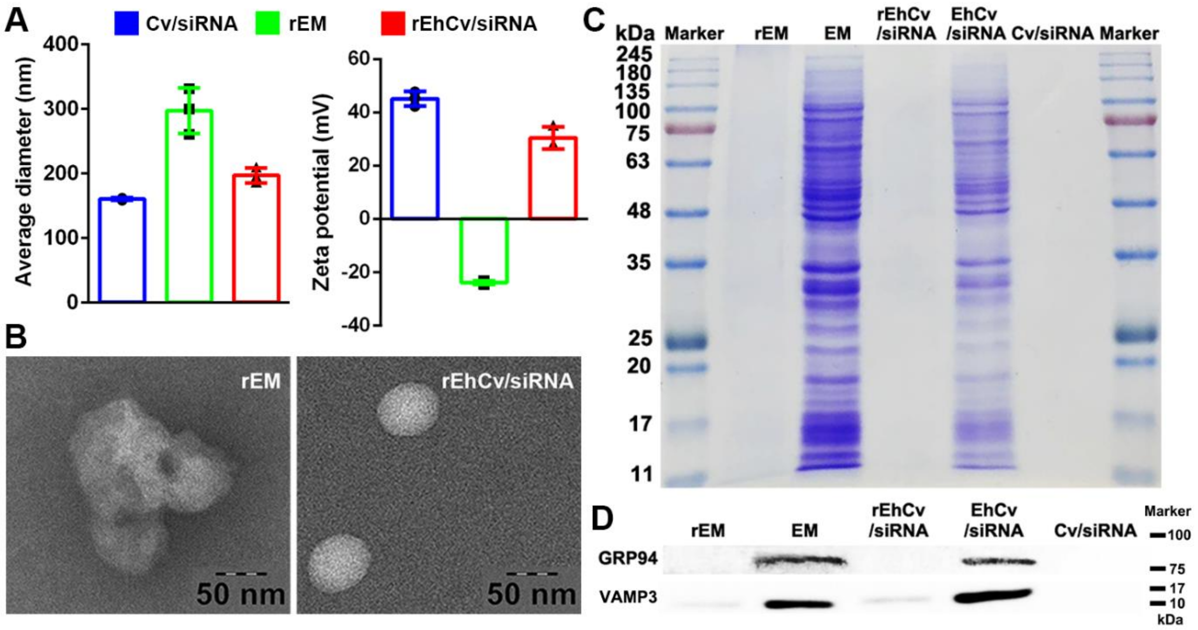

Supplementary Figure 7. Characterization of rEhCv/siRNA NPs whose rEM derived from MCF-7 cells. **A)** Particle sizes and zeta potentials of the Cv/siRNA NPs, rEM and rEhCv/siRNA NPs. **B)** TEM images of rEM and rEhCv/siRNA NPs. **C)** SDS-PAGE protein analysis. **D)** Western blotting analysis of GRP94 and VAMP3.

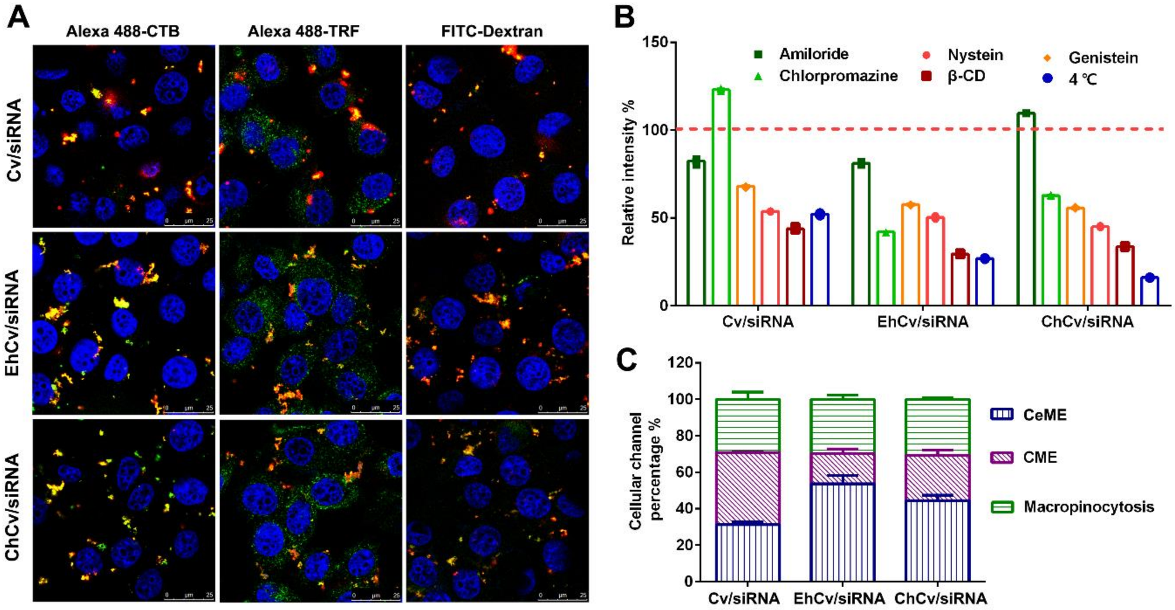

Supplementary Figure 8. The endocytosis pathway of three NPs on MCF-7 cells. **A**) Qualitative analysis of endocytosis pathways by confocal microscopy. The yellow dots represent the colocalization of pathway markers (green) and Cy5-siRNA (100 nM, red). **B**) Quantitative analysis of intracellular fluorescence intensities by flow cytometry after 4 h incubation of different NPs (FAM-labeled siRNA: 100 nM) with or without inhibitors (amiloride: 0.5 mM, chlorpromazine: 10 μg mL^{-1}, β-CD: 5 mM, genistein: 40 μg mL^{-1}, nystatin: 40 μg mL^{-1}). **C**) Channel ratios of three NPs in cellular uptake, which was calculated with the Pearson correlation coefficient of colocalization. The data were shown as mean ± SD (n = 3).

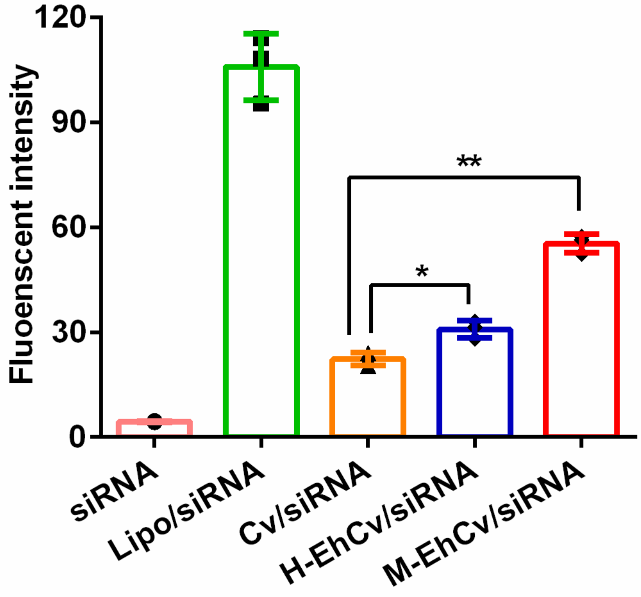

Supplementary Figure 9. Intracellular fluorescence intensities in A549 cells after treated with H-EhCv/siRNA NPs and M-EhCv/siRNA NPs which EM were isolated from HT-1080 and MCF-7 cells, respectively. The final concentration of FAM-labled siNC was 100 nM and the data were shown as mean ± SD (n = 3). *$p < 0.05$, **$p < 0.01$, t-test.

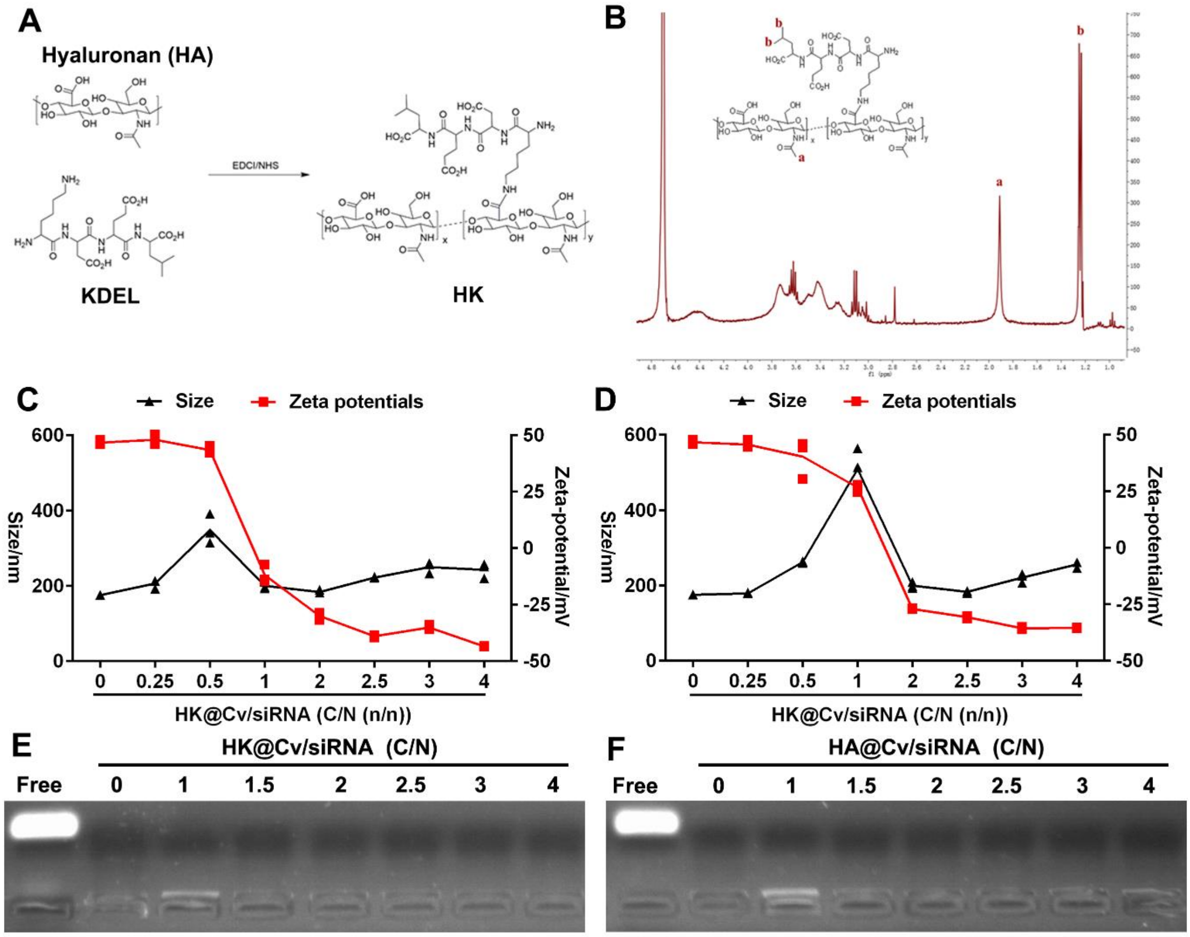

Supplementary Figure 10. Formulation optimization of HK@Cv/siRNA NPs and HA@Cv/siRNA NPs. **A**) The hyaluronan-KDEL graft polymer (HK) was successfully synthesized by conjugating the amine group of KDEL with carboxyl group of hyaluronic acid via EDC/NHS coupling technique. **B**) The ^{1}H-NMR spectrum of HK. **C**) and **D**) The optimization of HK@Cv/siRNA NPs and HA@Cv/siRNA NPs based on the size and zeta potentials measured by Zetasizer Nano ZS (siRNA: 100 nM, the data were shown as mean ± SD (n = 3)). **E**) and **F**) The siRNA loading capability of HK@Cv/siRNA NPs and HA@Cv/siRNA NPs with different molar ratios of C/N (C: carboxyl group in HK or HA, N: amidogen in DOTAP) detected by the gel retardation assay (siRNA: 1 μM, N/P=5/1).

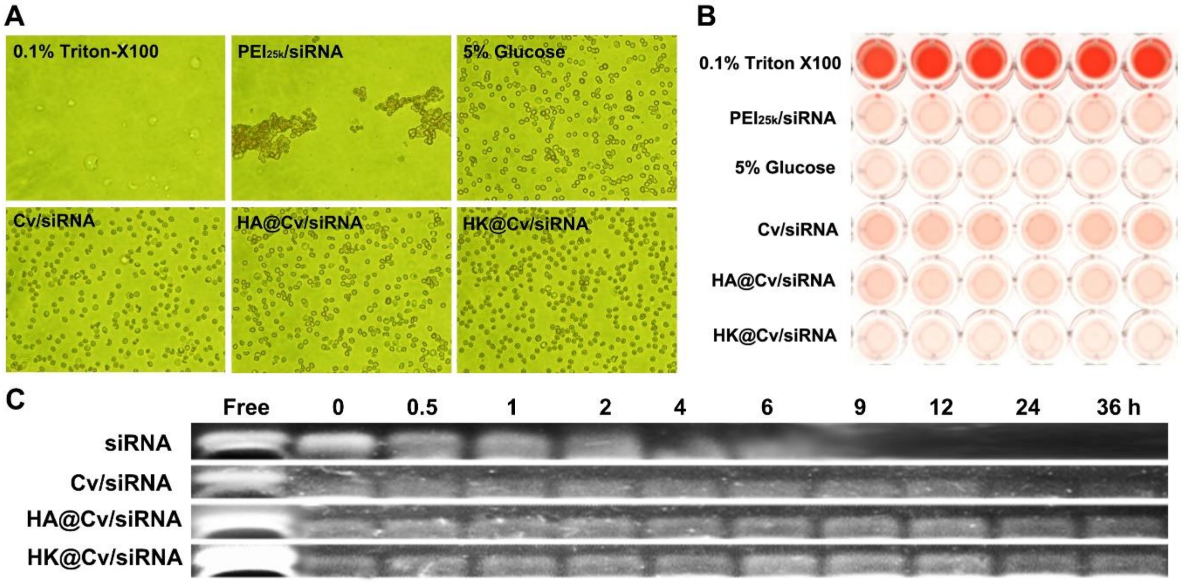

Supplementary Figure 11. Evaluation of safety and stability of HK@Cv/siRNA NPs. **A)** The hemagglutination test; **B)** The hemolysis activity. The final concentration of siNC was 100 nM. **C)** The protective efficacy of various NPs on siRNA detected by gel retardation assay (siRNA: 1μM).

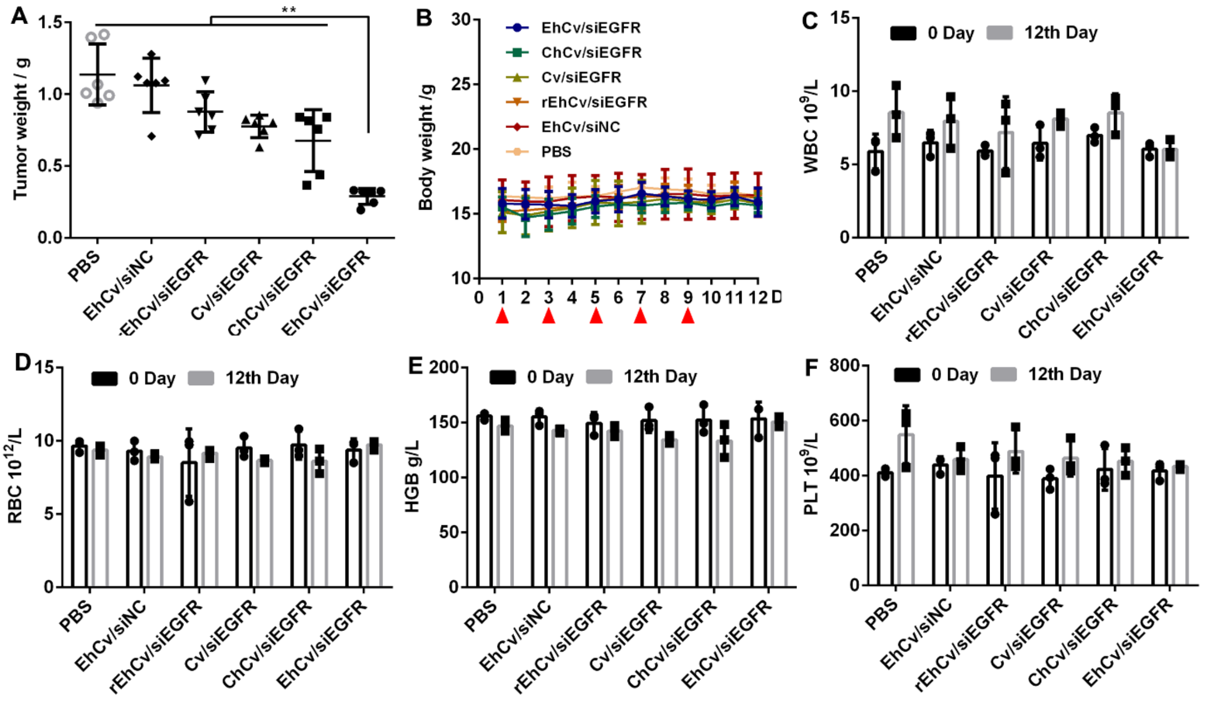

Supplementary Figure 12. Tumor weight and safety evaluation of different siRNA-loaded NPs on female BALB/c nude mice bearing MCF-7 tumors after peritumoral injection. A) Tumor weight at 12th day; B) the changes of body weight during the treatment (n = 6); C~F) examination of the changes of WBC, RBC, HGB and PLT in the serum of nude mice at the 0 and 12th day (n = 3). The data were shown as mean ± SD, ***p* < 0.01, ANOVA.

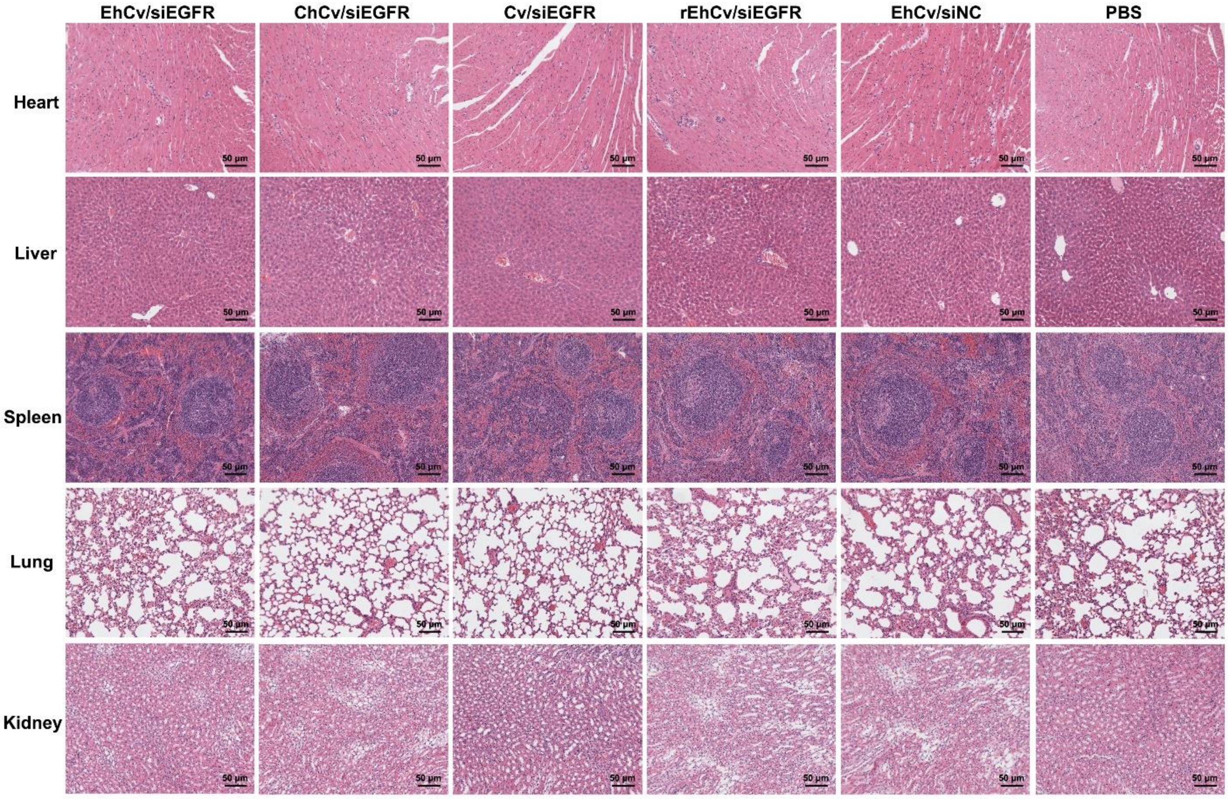

Supplementary Figure 13. Haematoxylin-eosin staining of tissue sections. The major organs were taken from the sacrificed nude mice at the end of drug administration.
