# Supplementary figures and images for: Regulating intracellular fate of siRNA by endoplasmic reticulum membrane-decorated hybrid nanoplexes
Source: Nat Commun. 2019 Jun 20;10:2702. doi: 10.1038/s41467-019-10562-w (PMC6586638; doi:10.1038/s41467-019-10562-w)

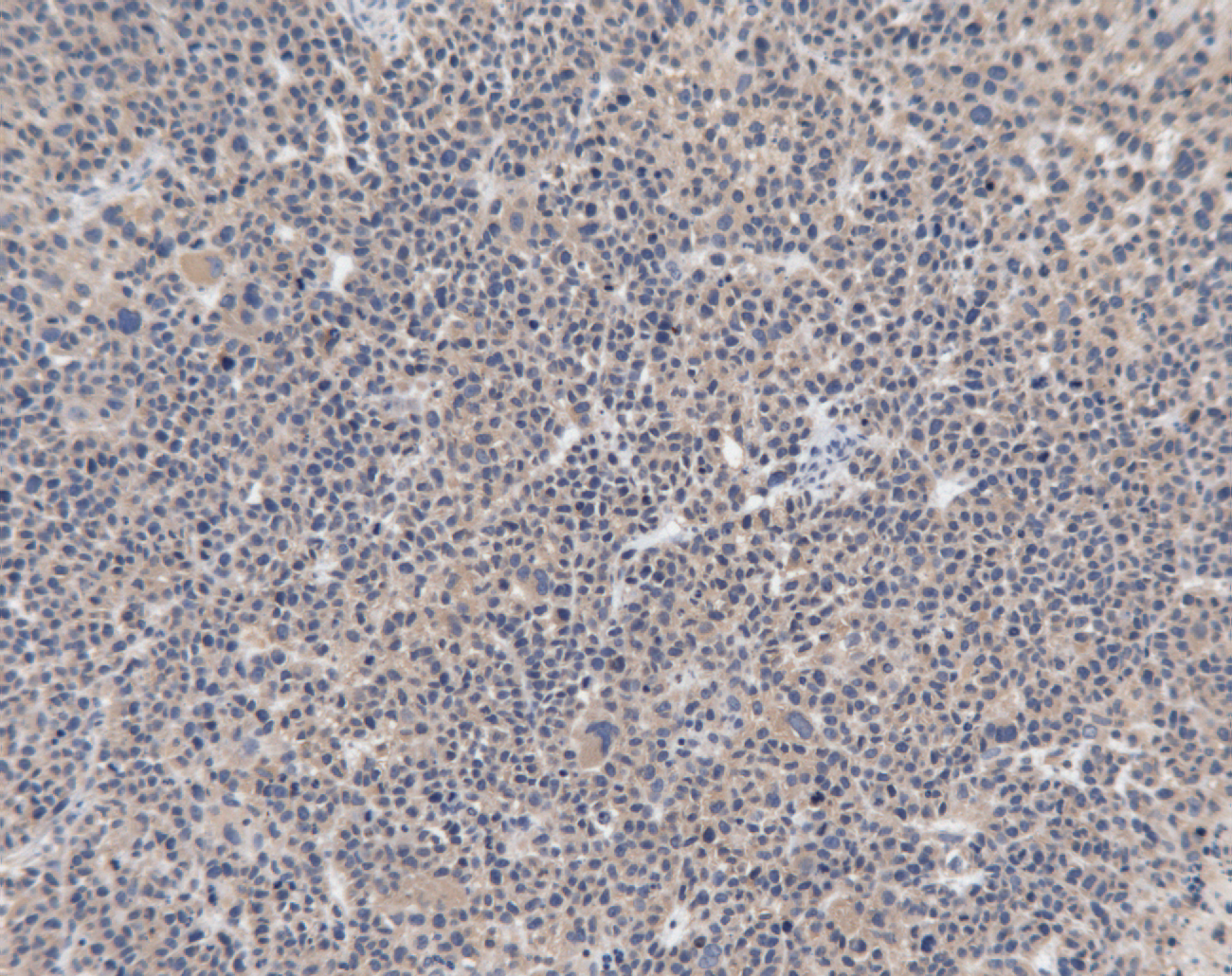

Supplement: Supplementary file 3 — Source Data [file 41467_2019_10562_MOESM3_ESM.zip › Source Data/Fig.8/Fig.8D/Fig.8D/ChCvsiEGFR.tif]

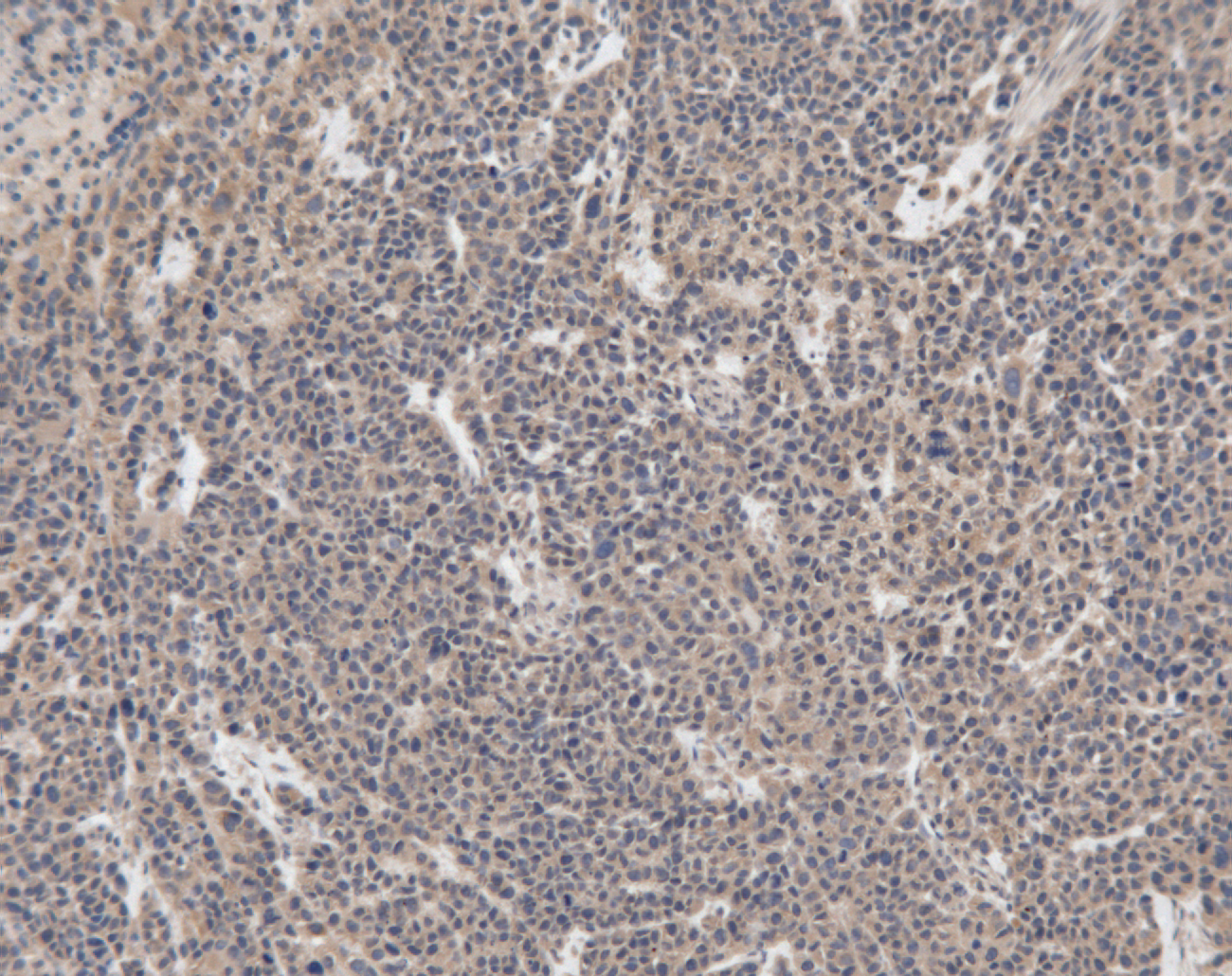

Supplement: Supplementary file 3 — Source Data [file 41467_2019_10562_MOESM3_ESM.zip › Source Data/Fig.8/Fig.8D/Fig.8D/CvsiEGFR.tif]

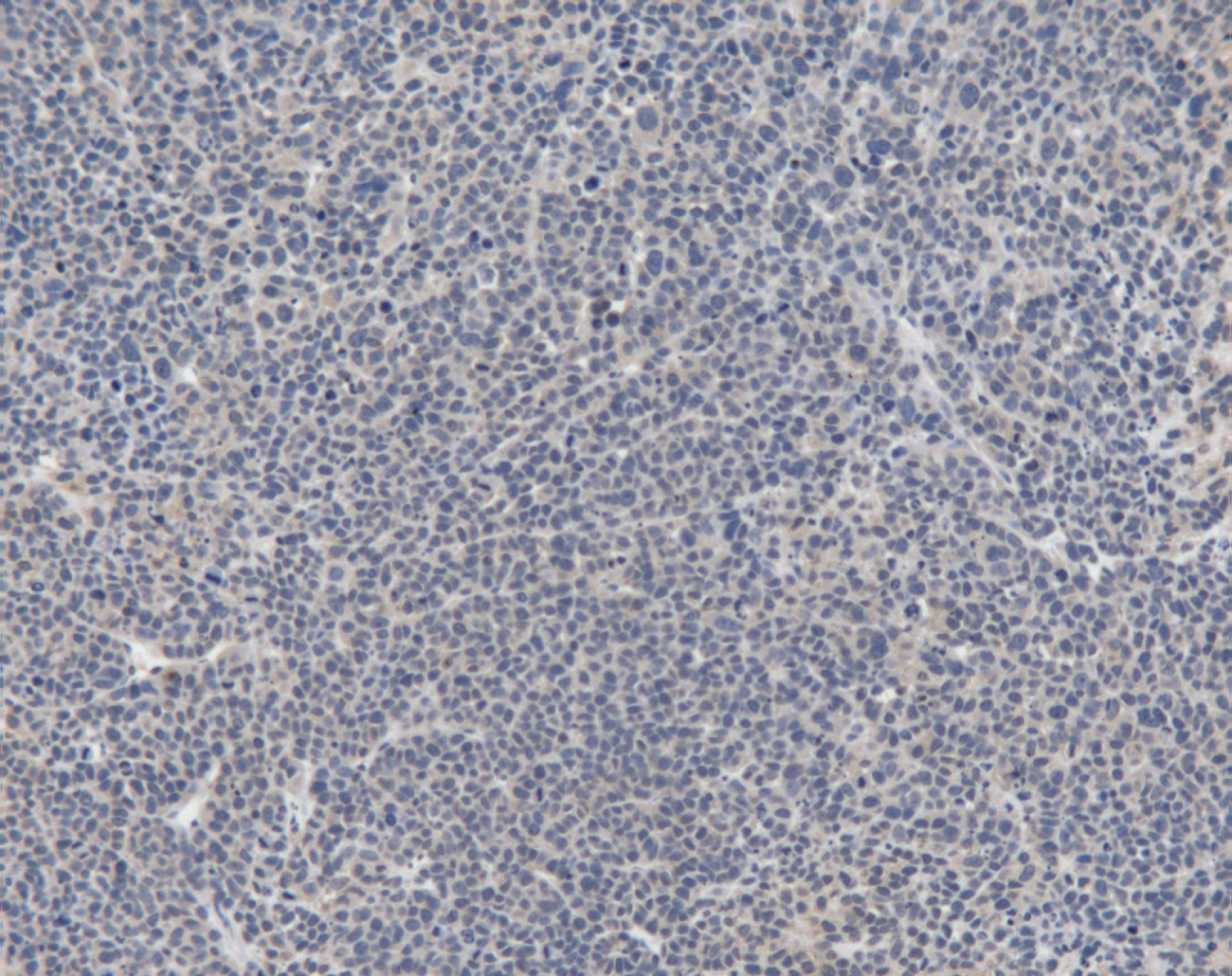

Supplement: Supplementary file 3 — Source Data [file 41467_2019_10562_MOESM3_ESM.zip › Source Data/Fig.8/Fig.8D/Fig.8D/EhCvsiEGFR.tif]

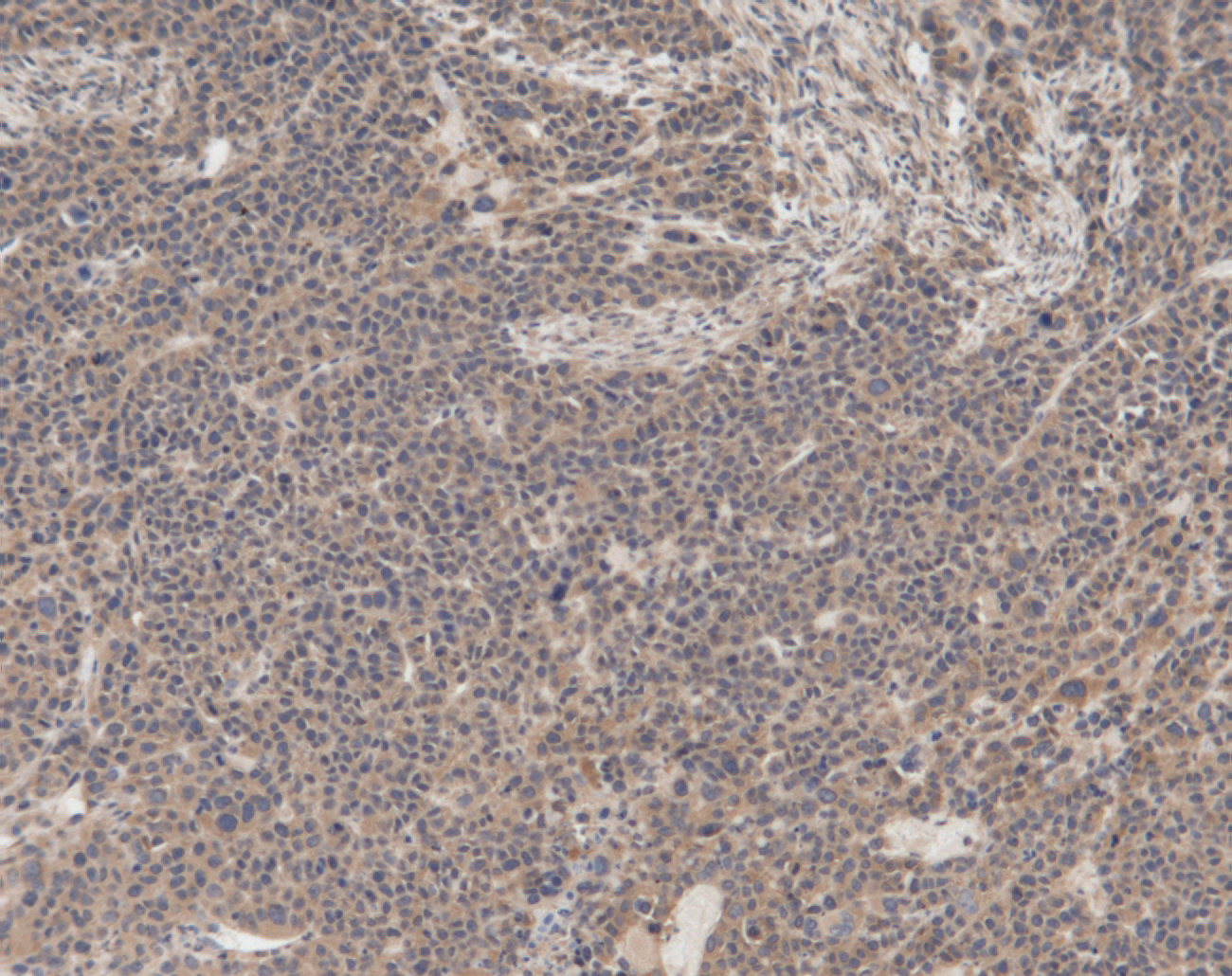

Supplement: Supplementary file 3 — Source Data [file 41467_2019_10562_MOESM3_ESM.zip › Source Data/Fig.8/Fig.8D/Fig.8D/EhCvsiNC.tif]

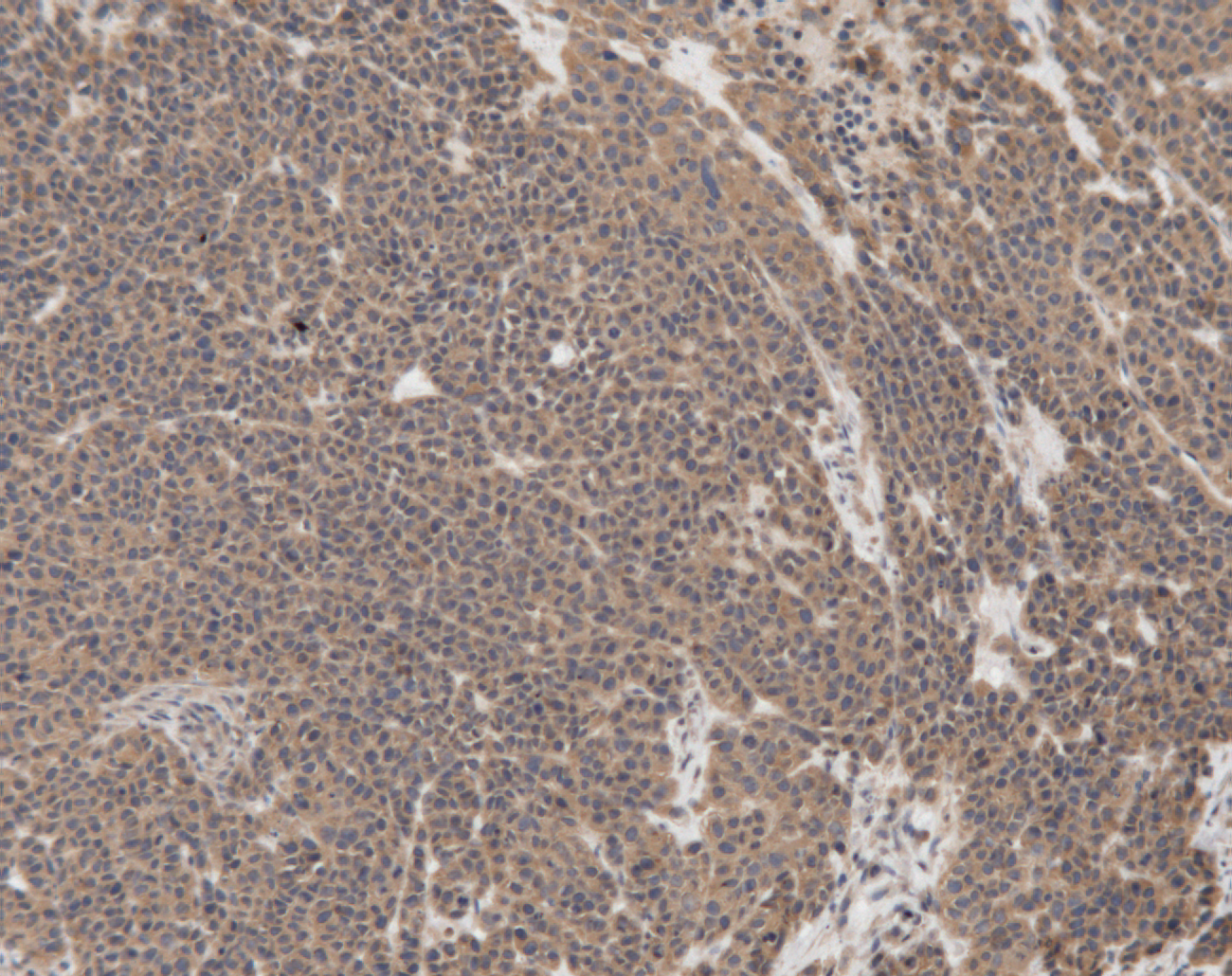

Supplement: Supplementary file 3 — Source Data [file 41467_2019_10562_MOESM3_ESM.zip › Source Data/Fig.8/Fig.8D/Fig.8D/PBS.tif]

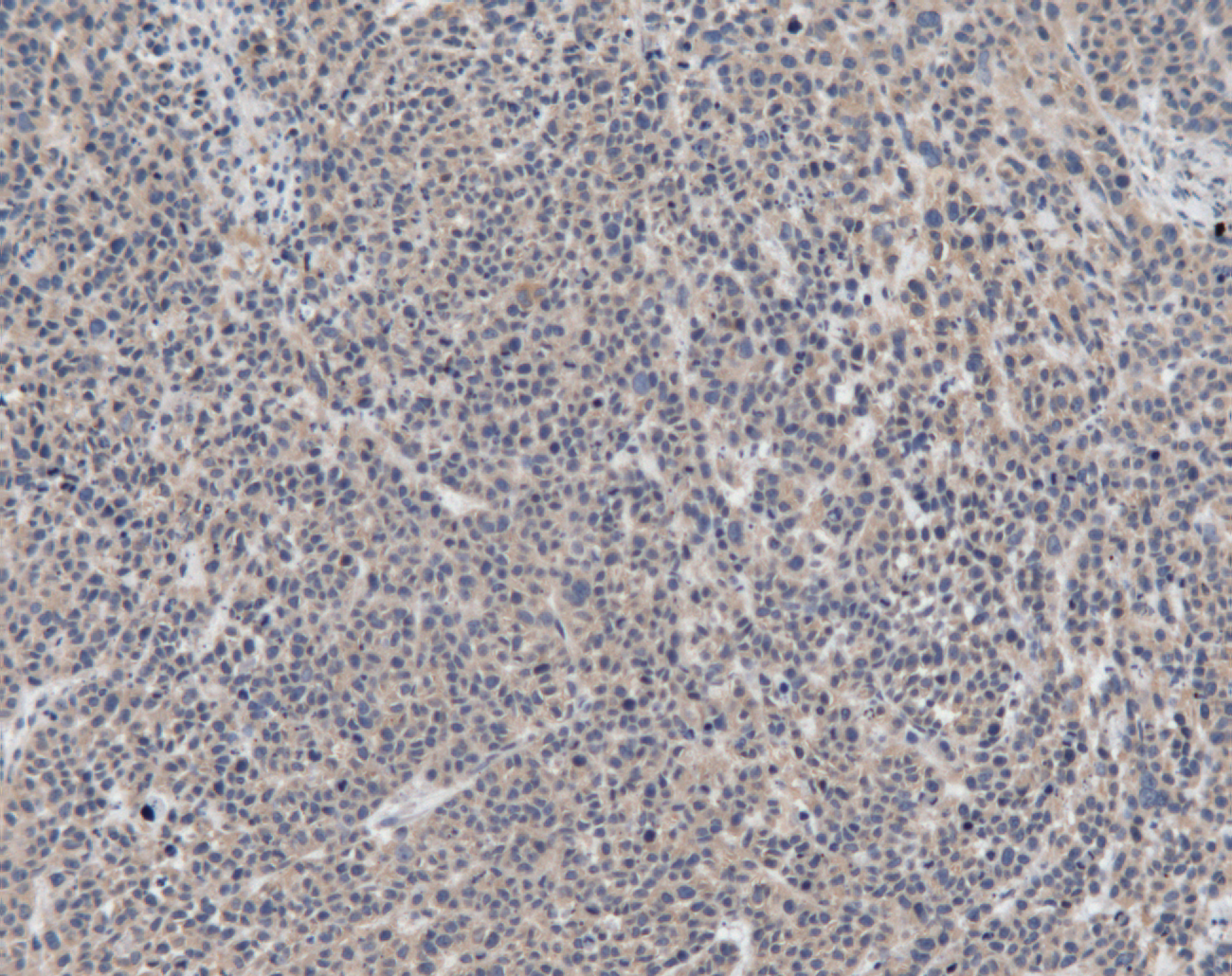

Supplement: Supplementary file 3 — Source Data [file 41467_2019_10562_MOESM3_ESM.zip › Source Data/Fig.8/Fig.8D/Fig.8D/rEhCvsiEGFR.tif]

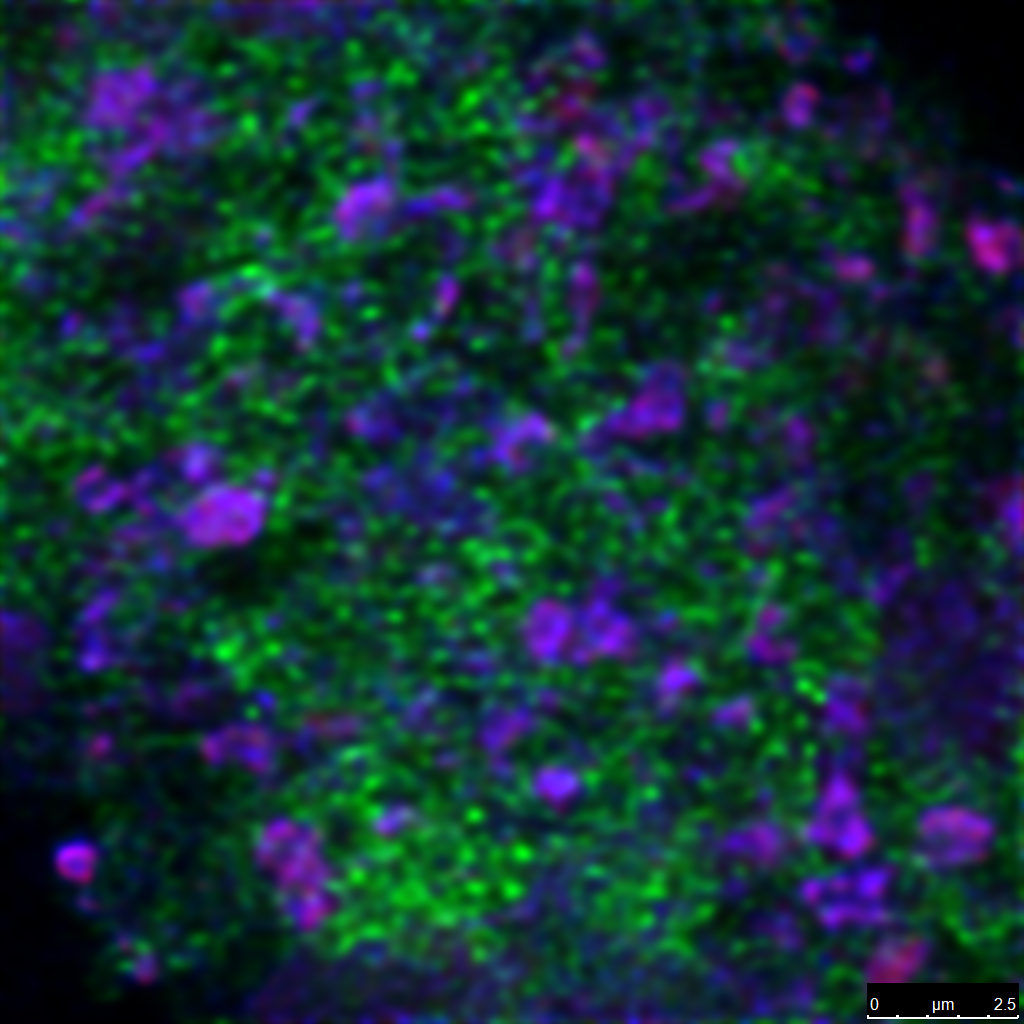

Supplement: Supplementary file 3 — Source Data [file 41467_2019_10562_MOESM3_ESM.zip › Source Data/Supplementary Fig.2/Supplementary Fig.2_Image001.tif]

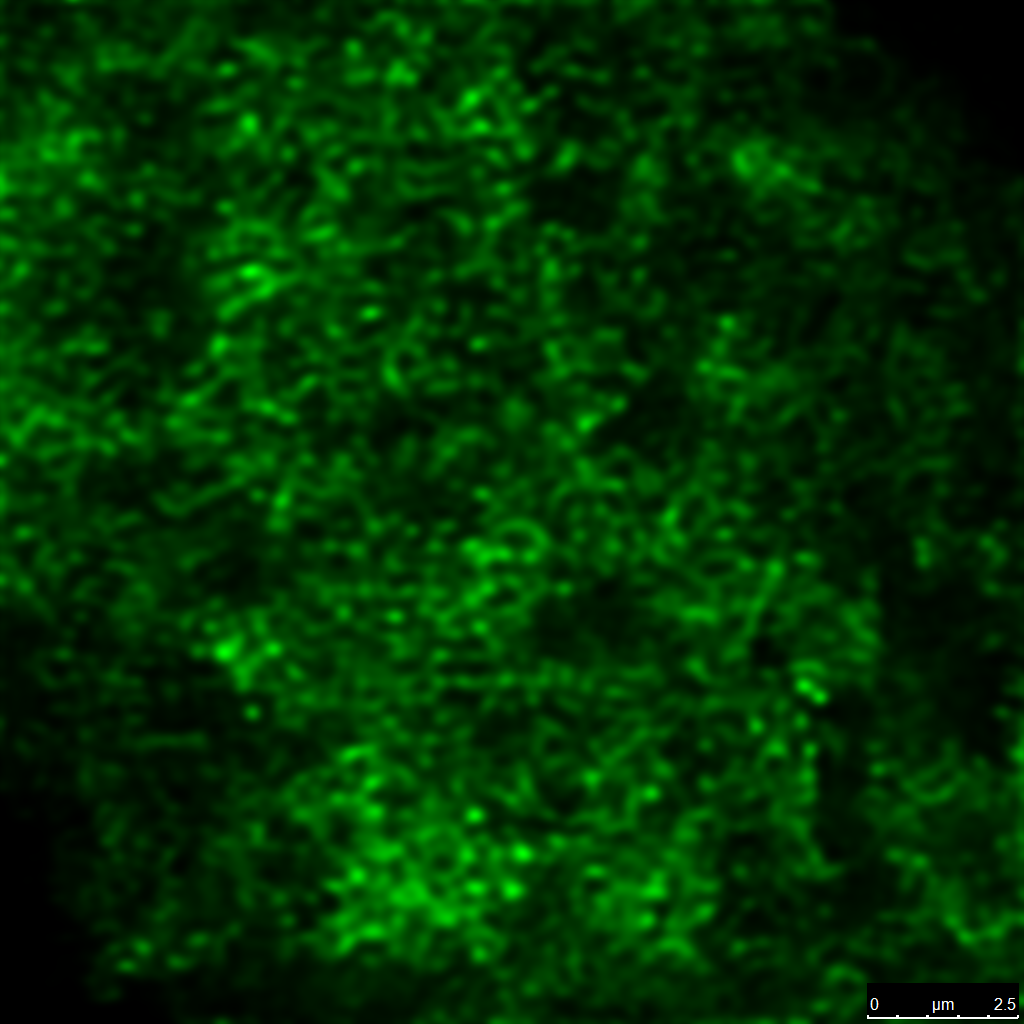

Supplement: Supplementary file 3 — Source Data [file 41467_2019_10562_MOESM3_ESM.zip › Source Data/Supplementary Fig.2/Supplementary Fig.2_Image001_ch00.tif]

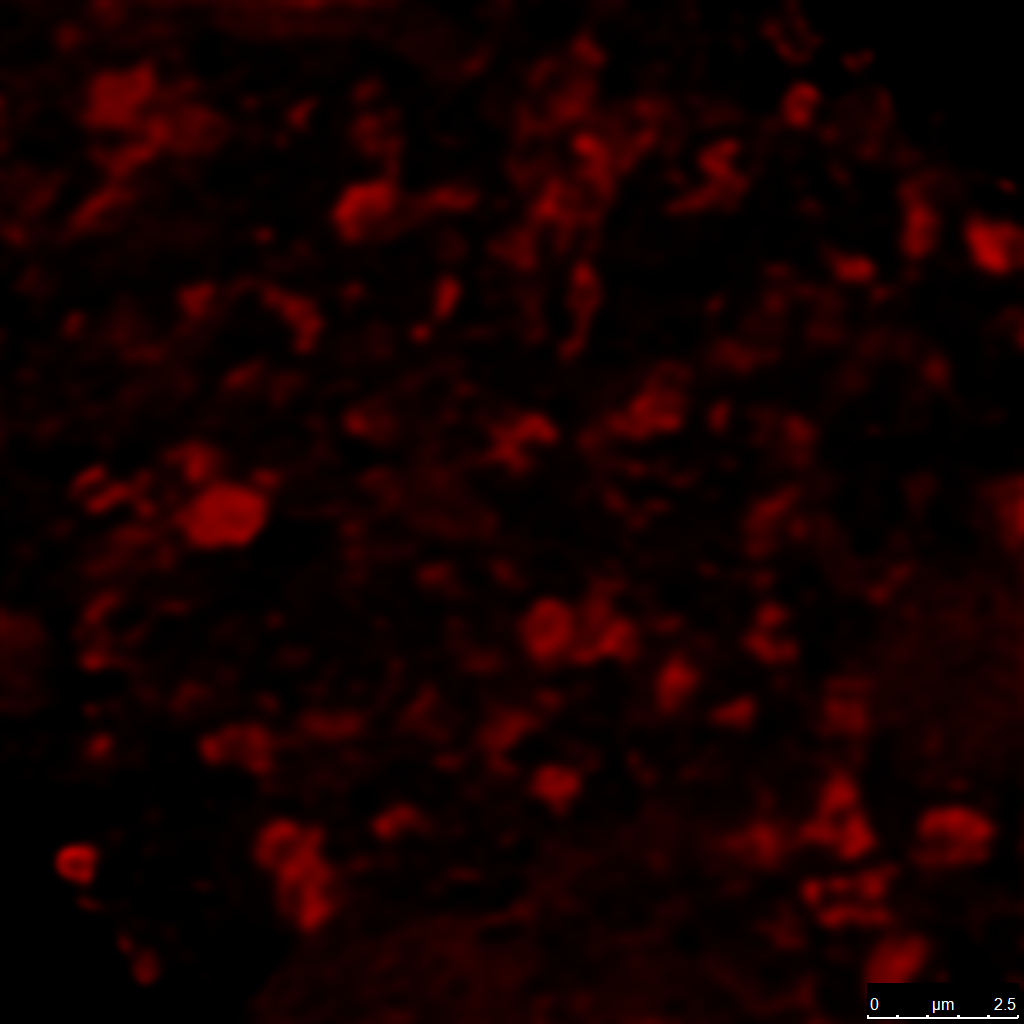

Supplement: Supplementary file 3 — Source Data [file 41467_2019_10562_MOESM3_ESM.zip › Source Data/Supplementary Fig.2/Supplementary Fig.2_Image001_ch01.tif]

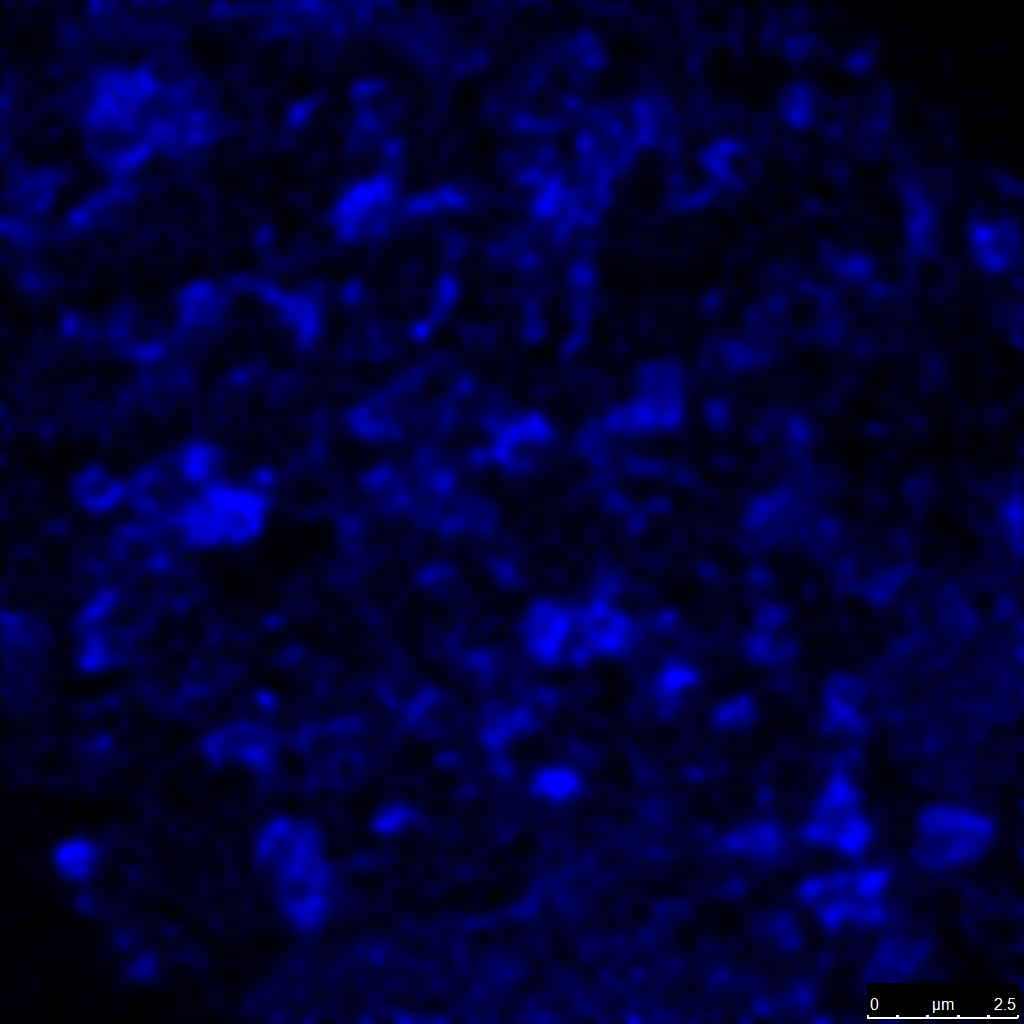

Supplement: Supplementary file 3 — Source Data [file 41467_2019_10562_MOESM3_ESM.zip › Source Data/Supplementary Fig.2/Supplementary Fig.2_Image001_ch02.tif]

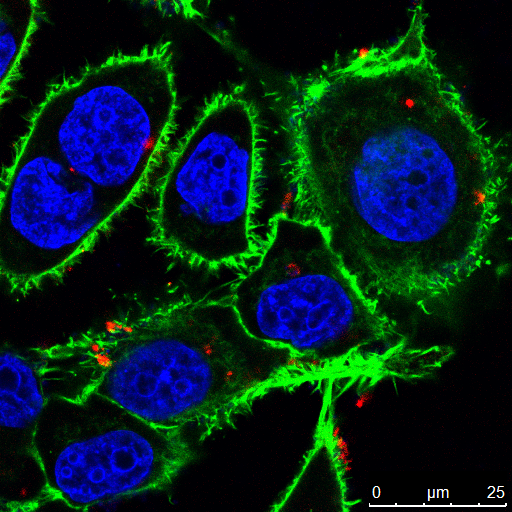

Supplement: Supplementary file 3 — Source Data [file 41467_2019_10562_MOESM3_ESM.zip › Source Data/Supplementary Fig.5/Cellular uptake in MCF-7 cancer cells_ChCvsiRNA.tif]

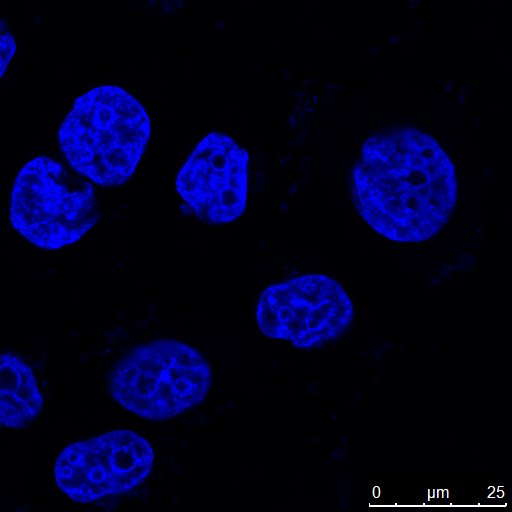

Supplement: Supplementary file 3 — Source Data [file 41467_2019_10562_MOESM3_ESM.zip › Source Data/Supplementary Fig.5/Cellular uptake in MCF-7 cancer cells_ChCvsiRNA_ch00.tif]

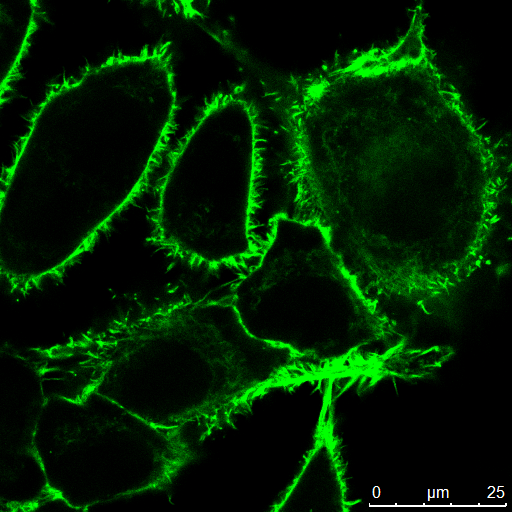

Supplement: Supplementary file 3 — Source Data [file 41467_2019_10562_MOESM3_ESM.zip › Source Data/Supplementary Fig.5/Cellular uptake in MCF-7 cancer cells_ChCvsiRNA_ch01.tif]

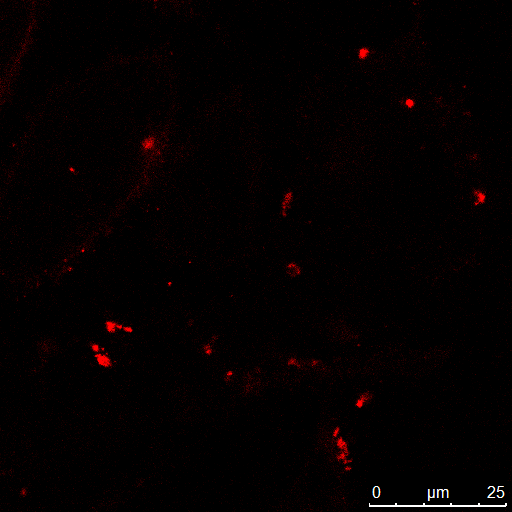

Supplement: Supplementary file 3 — Source Data [file 41467_2019_10562_MOESM3_ESM.zip › Source Data/Supplementary Fig.5/Cellular uptake in MCF-7 cancer cells_ChCvsiRNA_ch02.tif]

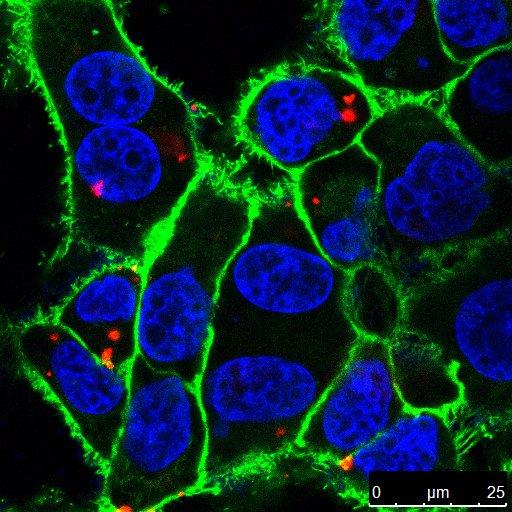

Supplement: Supplementary file 3 — Source Data [file 41467_2019_10562_MOESM3_ESM.zip › Source Data/Supplementary Fig.5/Cellular uptake in MCF-7 cancer cells_CvsiRNA.tif]

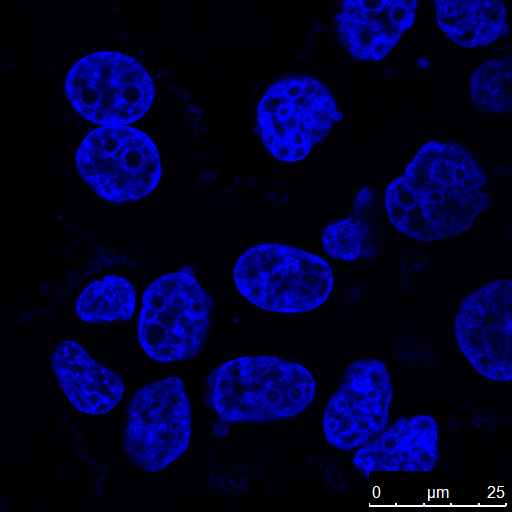

Supplement: Supplementary file 3 — Source Data [file 41467_2019_10562_MOESM3_ESM.zip › Source Data/Supplementary Fig.5/Cellular uptake in MCF-7 cancer cells_CvsiRNA_ch00.tif]

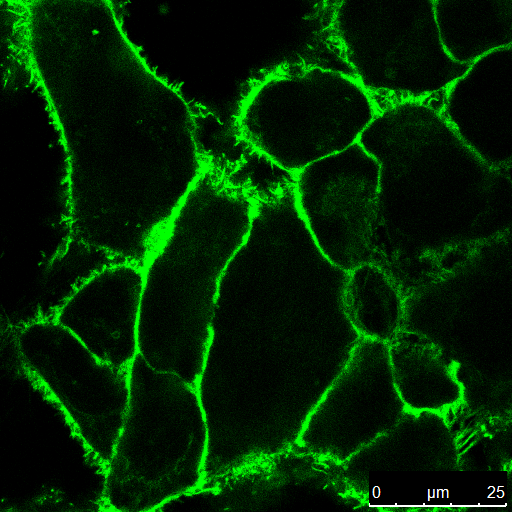

Supplement: Supplementary file 3 — Source Data [file 41467_2019_10562_MOESM3_ESM.zip › Source Data/Supplementary Fig.5/Cellular uptake in MCF-7 cancer cells_CvsiRNA_ch01.tif]

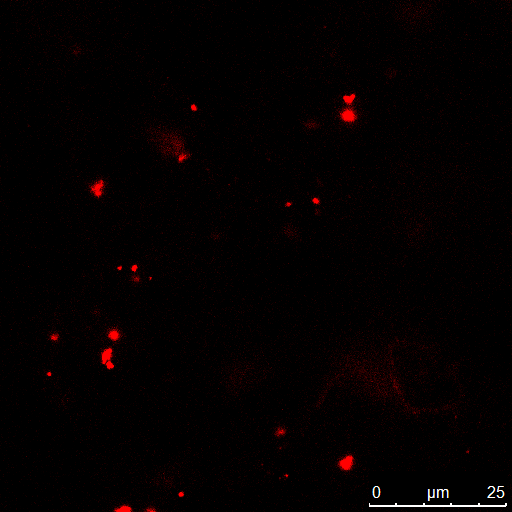

Supplement: Supplementary file 3 — Source Data [file 41467_2019_10562_MOESM3_ESM.zip › Source Data/Supplementary Fig.5/Cellular uptake in MCF-7 cancer cells_CvsiRNA_ch02.tif]

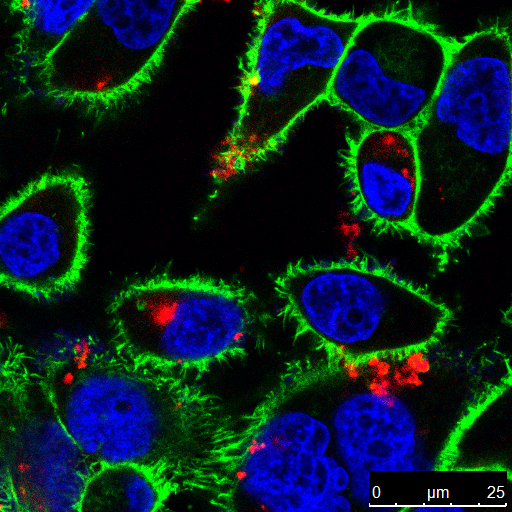

Supplement: Supplementary file 3 — Source Data [file 41467_2019_10562_MOESM3_ESM.zip › Source Data/Supplementary Fig.5/Cellular uptake in MCF-7 cancer cells_EhCvsiRNA.tif]

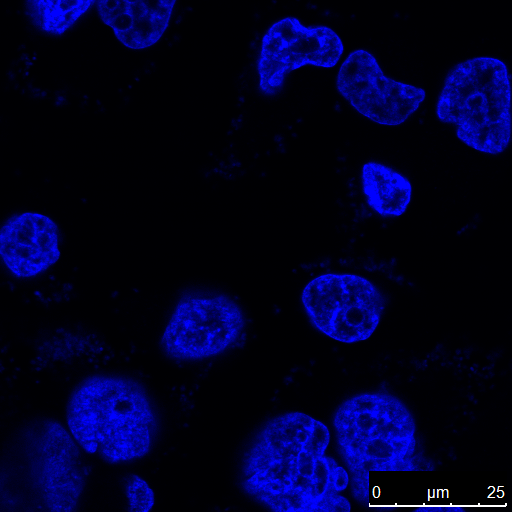

Supplement: Supplementary file 3 — Source Data [file 41467_2019_10562_MOESM3_ESM.zip › Source Data/Supplementary Fig.5/Cellular uptake in MCF-7 cancer cells_EhCvsiRNA_ch00.tif]

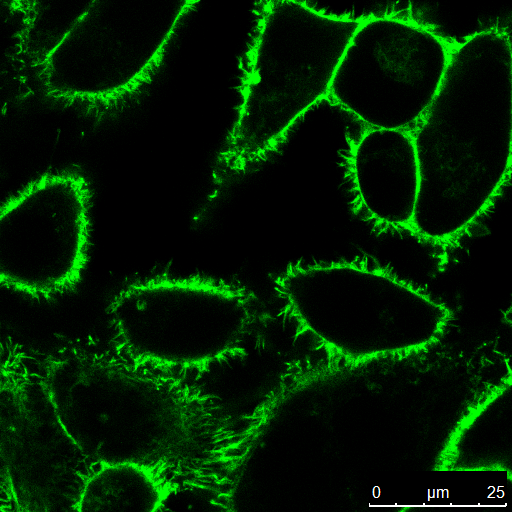

Supplement: Supplementary file 3 — Source Data [file 41467_2019_10562_MOESM3_ESM.zip › Source Data/Supplementary Fig.5/Cellular uptake in MCF-7 cancer cells_EhCvsiRNA_ch01.tif]

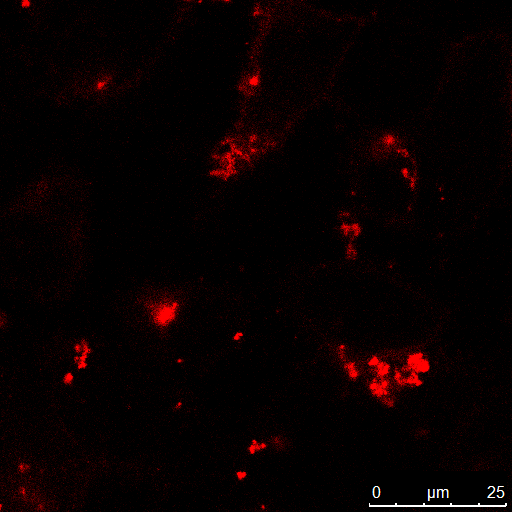

Supplement: Supplementary file 3 — Source Data [file 41467_2019_10562_MOESM3_ESM.zip › Source Data/Supplementary Fig.5/Cellular uptake in MCF-7 cancer cells_EhCvsiRNA_ch02.tif]

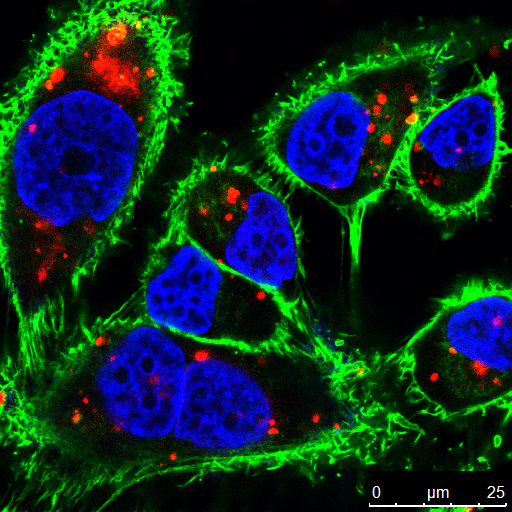

Supplement: Supplementary file 3 — Source Data [file 41467_2019_10562_MOESM3_ESM.zip › Source Data/Supplementary Fig.5/Cellular uptake in MCF-7 cancer cells_LiposiRNA.tif]

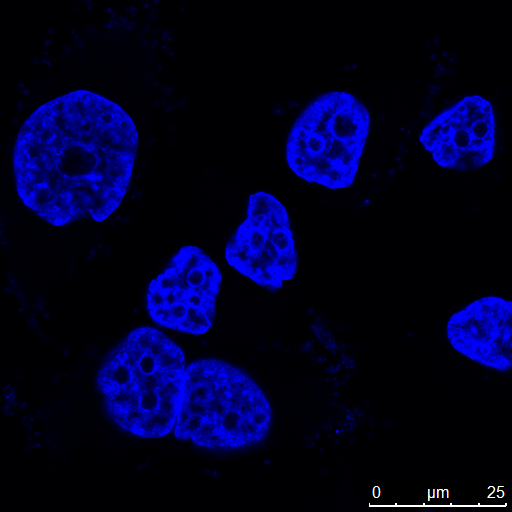

Supplement: Supplementary file 3 — Source Data [file 41467_2019_10562_MOESM3_ESM.zip › Source Data/Supplementary Fig.5/Cellular uptake in MCF-7 cancer cells_LiposiRNA_ch00.tif]

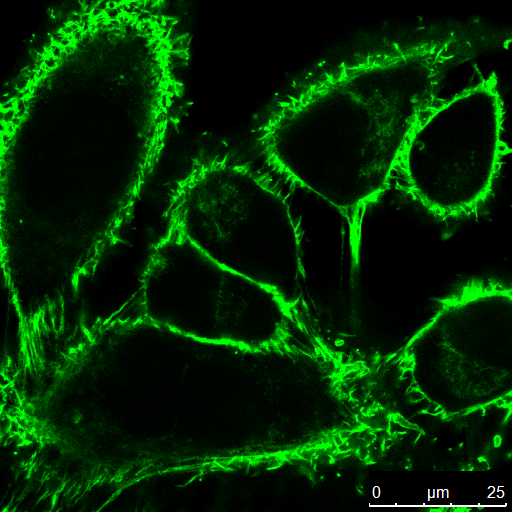

Supplement: Supplementary file 3 — Source Data [file 41467_2019_10562_MOESM3_ESM.zip › Source Data/Supplementary Fig.5/Cellular uptake in MCF-7 cancer cells_LiposiRNA_ch01.tif]

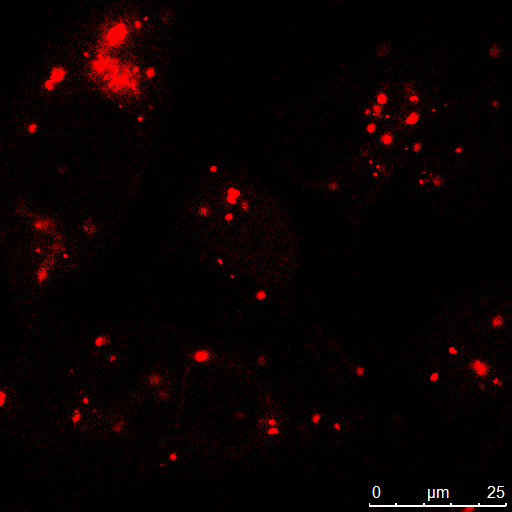

Supplement: Supplementary file 3 — Source Data [file 41467_2019_10562_MOESM3_ESM.zip › Source Data/Supplementary Fig.5/Cellular uptake in MCF-7 cancer cells_LiposiRNA_ch02.tif]

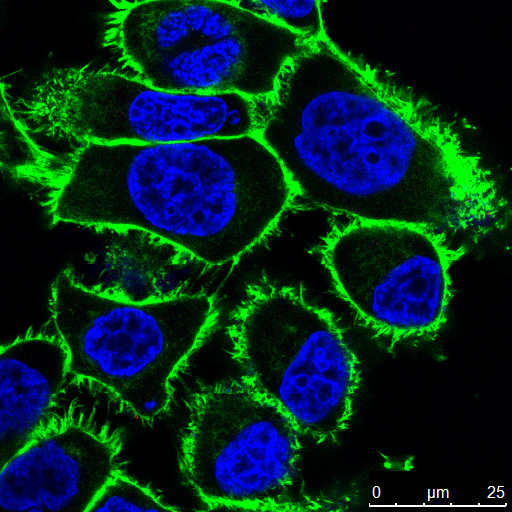

Supplement: Supplementary file 3 — Source Data [file 41467_2019_10562_MOESM3_ESM.zip › Source Data/Supplementary Fig.5/Cellular uptake in MCF-7 cancer cells_siRNA.tif]

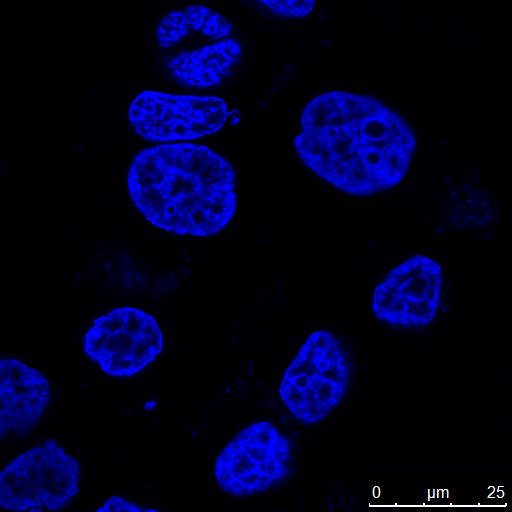

Supplement: Supplementary file 3 — Source Data [file 41467_2019_10562_MOESM3_ESM.zip › Source Data/Supplementary Fig.5/Cellular uptake in MCF-7 cancer cells_siRNA_ch00.tif]

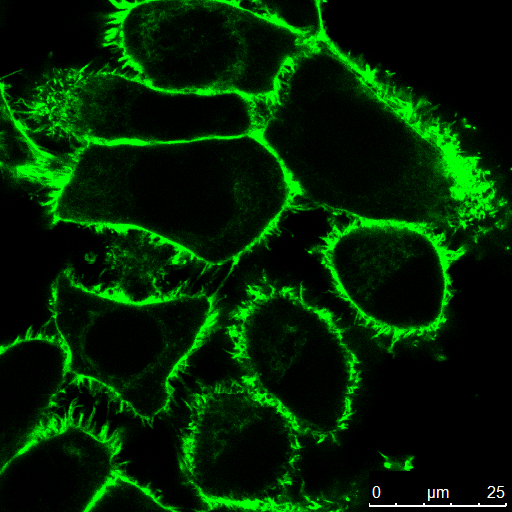

Supplement: Supplementary file 3 — Source Data [file 41467_2019_10562_MOESM3_ESM.zip › Source Data/Supplementary Fig.5/Cellular uptake in MCF-7 cancer cells_siRNA_ch01.tif]

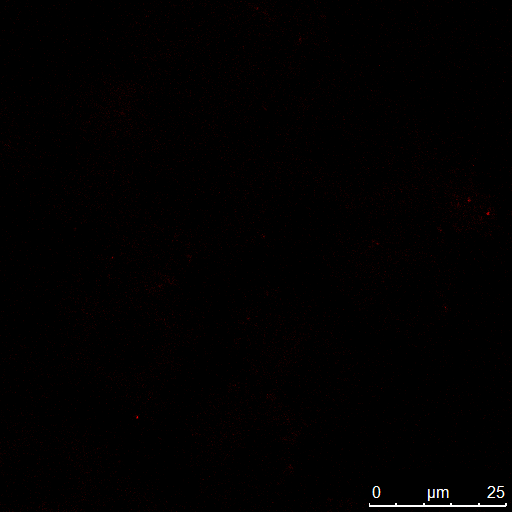

Supplement: Supplementary file 3 — Source Data [file 41467_2019_10562_MOESM3_ESM.zip › Source Data/Supplementary Fig.5/Cellular uptake in MCF-7 cancer cells_siRNA_ch02.tif]

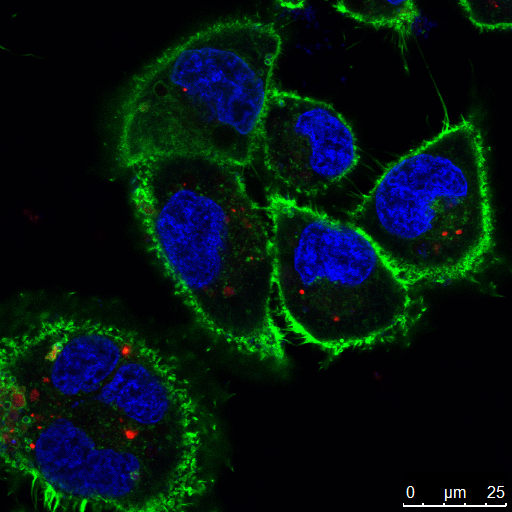

Supplement: Supplementary file 3 — Source Data [file 41467_2019_10562_MOESM3_ESM.zip › Source Data/Supplementary Fig.6/Cellular uptake in HT1080 cencer cells_ChCvsiRNA.tif]

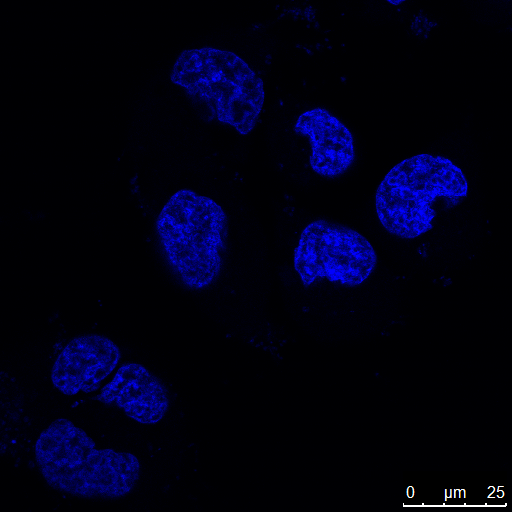

Supplement: Supplementary file 3 — Source Data [file 41467_2019_10562_MOESM3_ESM.zip › Source Data/Supplementary Fig.6/Cellular uptake in HT1080 cencer cells_ChCvsiRNA_ch00.tif]

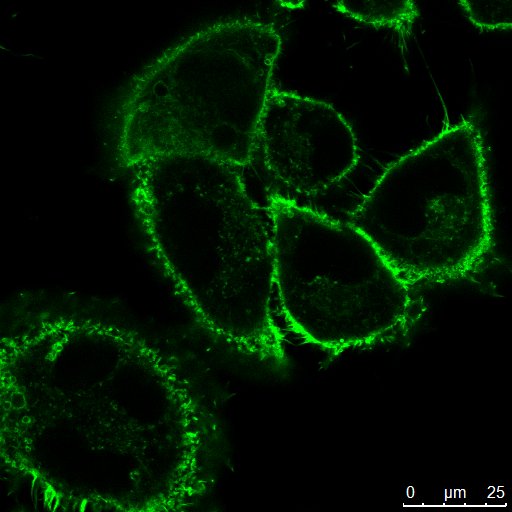

Supplement: Supplementary file 3 — Source Data [file 41467_2019_10562_MOESM3_ESM.zip › Source Data/Supplementary Fig.6/Cellular uptake in HT1080 cencer cells_ChCvsiRNA_ch01.tif]

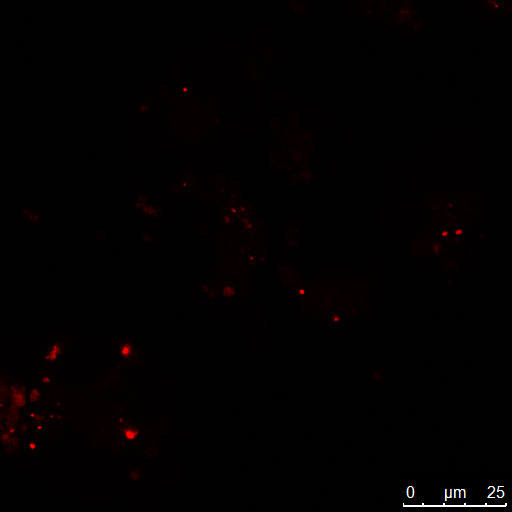

Supplement: Supplementary file 3 — Source Data [file 41467_2019_10562_MOESM3_ESM.zip › Source Data/Supplementary Fig.6/Cellular uptake in HT1080 cencer cells_ChCvsiRNA_ch02.tif]

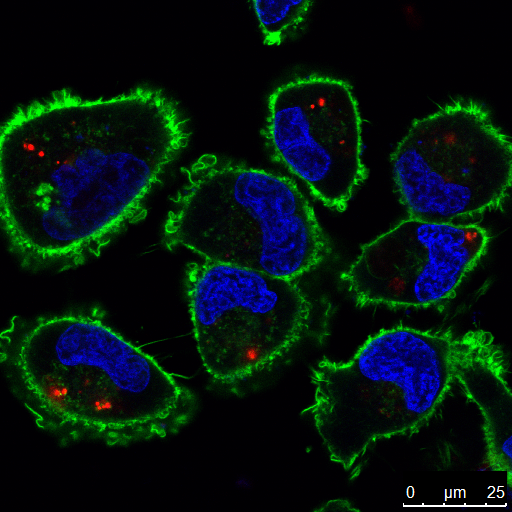

Supplement: Supplementary file 3 — Source Data [file 41467_2019_10562_MOESM3_ESM.zip › Source Data/Supplementary Fig.6/Cellular uptake in HT1080 cencer cells_CvsiRNA.tif]

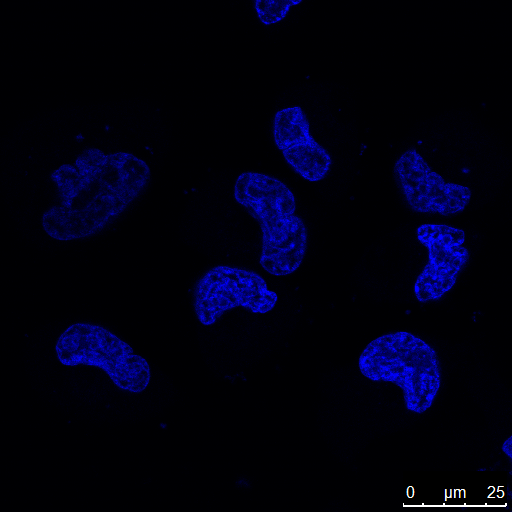

Supplement: Supplementary file 3 — Source Data [file 41467_2019_10562_MOESM3_ESM.zip › Source Data/Supplementary Fig.6/Cellular uptake in HT1080 cencer cells_CvsiRNA_ch00.tif]

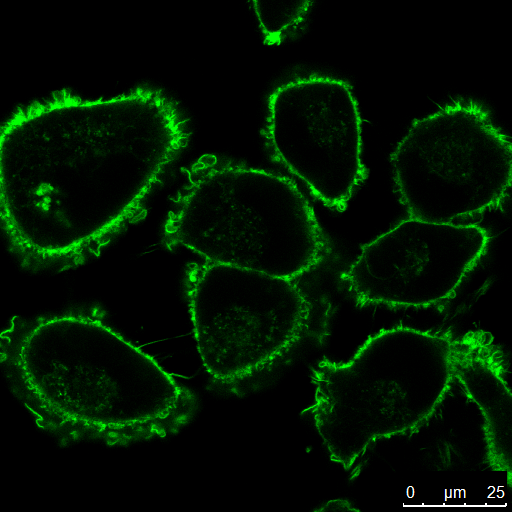

Supplement: Supplementary file 3 — Source Data [file 41467_2019_10562_MOESM3_ESM.zip › Source Data/Supplementary Fig.6/Cellular uptake in HT1080 cencer cells_CvsiRNA_ch01.tif]

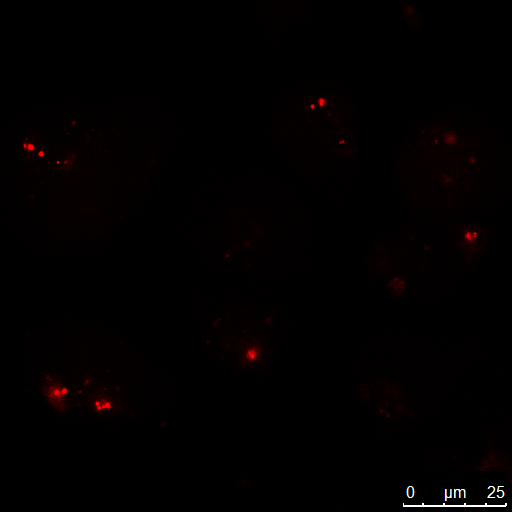

Supplement: Supplementary file 3 — Source Data [file 41467_2019_10562_MOESM3_ESM.zip › Source Data/Supplementary Fig.6/Cellular uptake in HT1080 cencer cells_CvsiRNA_ch02.tif]

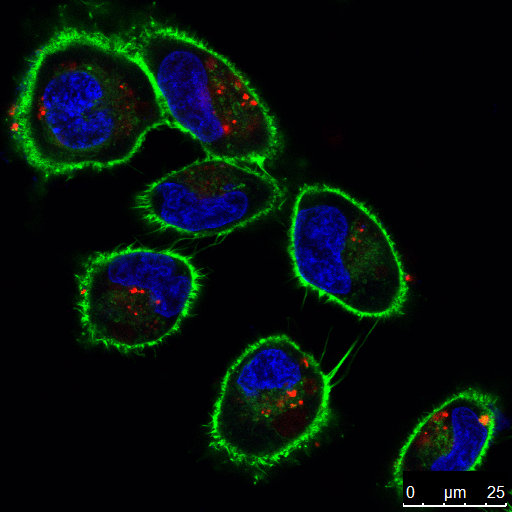

Supplement: Supplementary file 3 — Source Data [file 41467_2019_10562_MOESM3_ESM.zip › Source Data/Supplementary Fig.6/Cellular uptake in HT1080 cencer cells_EhCvsiRNA.tif]

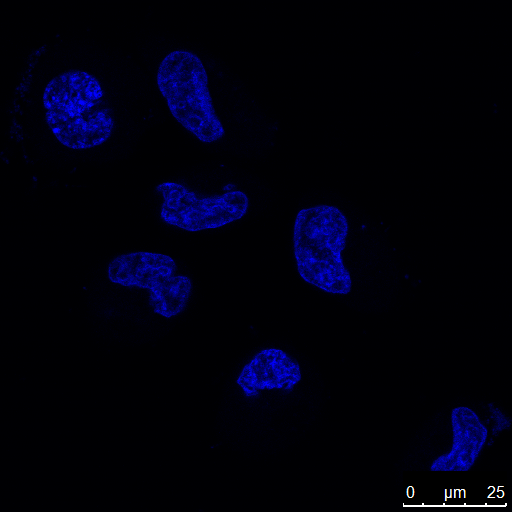

Supplement: Supplementary file 3 — Source Data [file 41467_2019_10562_MOESM3_ESM.zip › Source Data/Supplementary Fig.6/Cellular uptake in HT1080 cencer cells_EhCvsiRNA_ch00.tif]

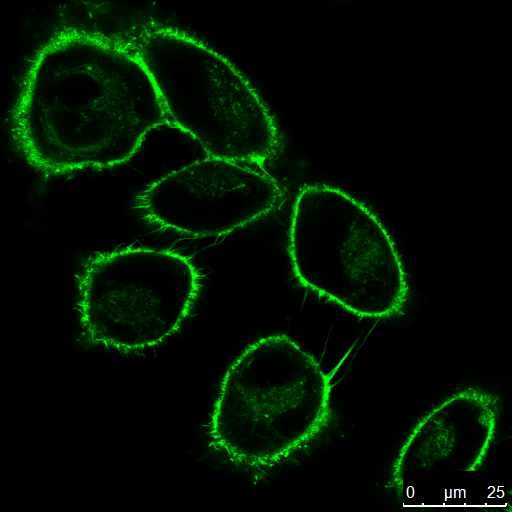

Supplement: Supplementary file 3 — Source Data [file 41467_2019_10562_MOESM3_ESM.zip › Source Data/Supplementary Fig.6/Cellular uptake in HT1080 cencer cells_EhCvsiRNA_ch01.tif]

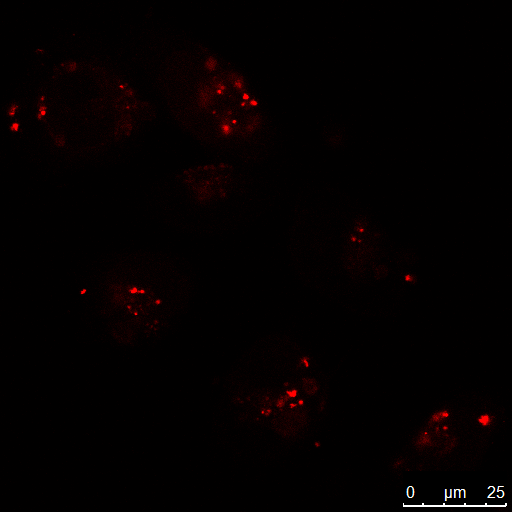

Supplement: Supplementary file 3 — Source Data [file 41467_2019_10562_MOESM3_ESM.zip › Source Data/Supplementary Fig.6/Cellular uptake in HT1080 cencer cells_EhCvsiRNA_ch02.tif]

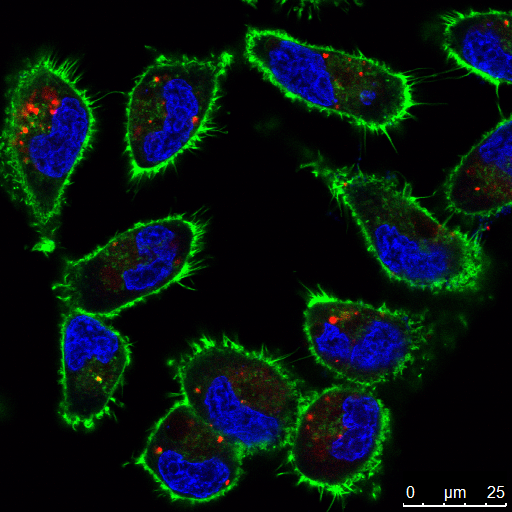

Supplement: Supplementary file 3 — Source Data [file 41467_2019_10562_MOESM3_ESM.zip › Source Data/Supplementary Fig.6/Cellular uptake in HT1080 cencer cells_LiposiRNA.tif]

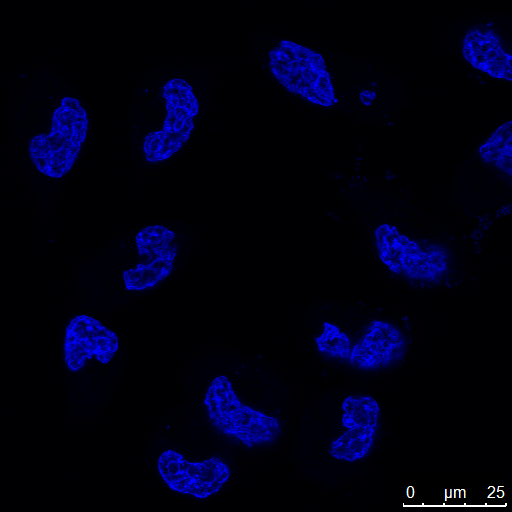

Supplement: Supplementary file 3 — Source Data [file 41467_2019_10562_MOESM3_ESM.zip › Source Data/Supplementary Fig.6/Cellular uptake in HT1080 cencer cells_LiposiRNA_ch00.tif]

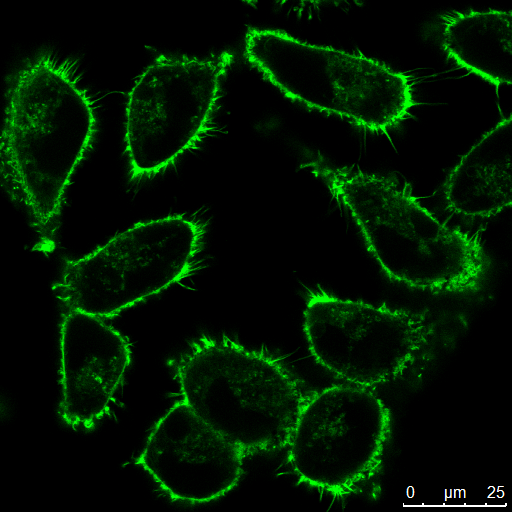

Supplement: Supplementary file 3 — Source Data [file 41467_2019_10562_MOESM3_ESM.zip › Source Data/Supplementary Fig.6/Cellular uptake in HT1080 cencer cells_LiposiRNA_ch01.tif]

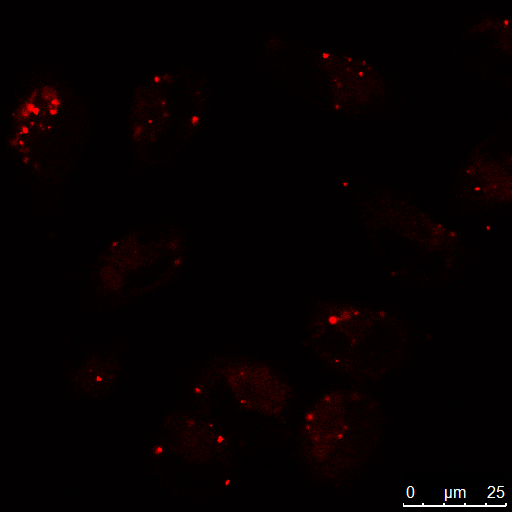

Supplement: Supplementary file 3 — Source Data [file 41467_2019_10562_MOESM3_ESM.zip › Source Data/Supplementary Fig.6/Cellular uptake in HT1080 cencer cells_LiposiRNA_ch02.tif]

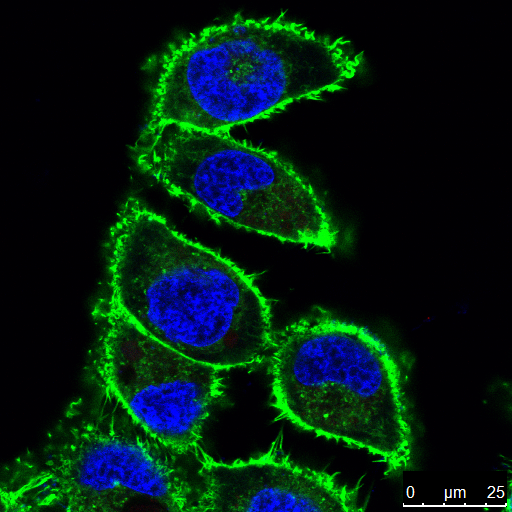

Supplement: Supplementary file 3 — Source Data [file 41467_2019_10562_MOESM3_ESM.zip › Source Data/Supplementary Fig.6/Cellular uptake in HT1080 cencer cells_siRNA.tif]

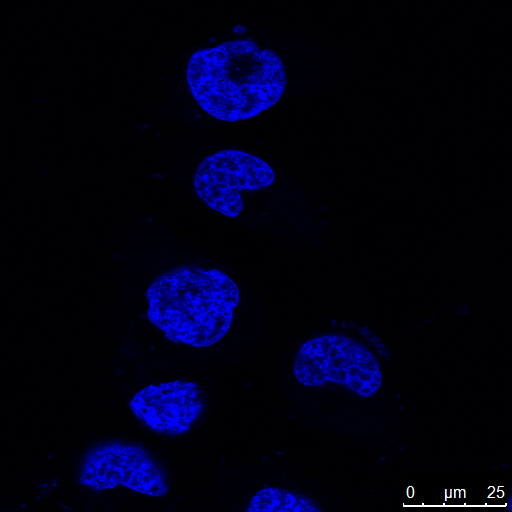

Supplement: Supplementary file 3 — Source Data [file 41467_2019_10562_MOESM3_ESM.zip › Source Data/Supplementary Fig.6/Cellular uptake in HT1080 cencer cells_siRNA_ch00.tif]

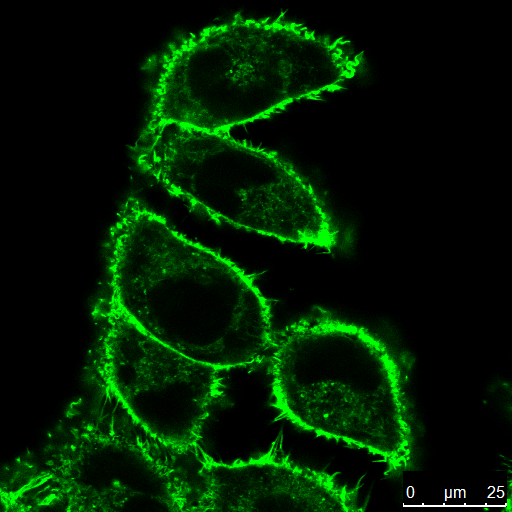

Supplement: Supplementary file 3 — Source Data [file 41467_2019_10562_MOESM3_ESM.zip › Source Data/Supplementary Fig.6/Cellular uptake in HT1080 cencer cells_siRNA_ch01.tif]

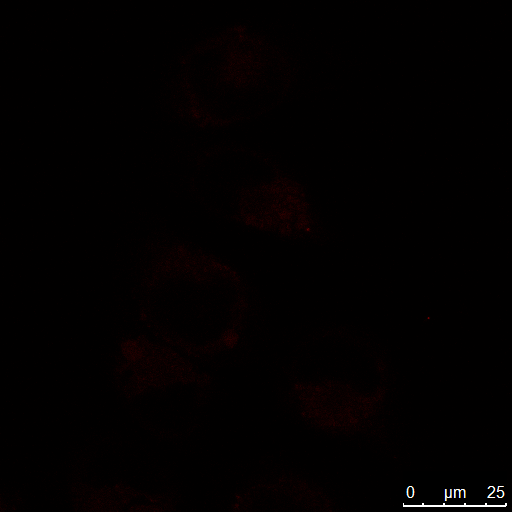

Supplement: Supplementary file 3 — Source Data [file 41467_2019_10562_MOESM3_ESM.zip › Source Data/Supplementary Fig.6/Cellular uptake in HT1080 cencer cells_siRNA_ch02.tif]
